# Characterization of Secondary Metabolites from Purple *Ipomoea batatas* Leaves and Their Effects on Glucose Uptake

**DOI:** 10.3390/molecules21060745

**Published:** 2016-06-08

**Authors:** Chia-Lin Lee, Shou-Lun Lee, Chao-Jung Chen, Hsin-Chun Chen, Ming-Ching Kao, Chuan-Hao Liu, Jau-Yang Chen, Yen-Ting Lai, Yang-Chang Wu

**Affiliations:** 1 Department of Cosmeceutics, China Medical University, Taichung 40402, Taiwan; chlilee@mail.cmu.edu.tw (C.-L.L.); c0706@mail.cmu.edu.tw (H.-C.C.); 2 Chinese Medicine Research and Development Center, China Medical University Hospital, Taichung 40447, Taiwan; bc00025@yahoo.com.tw (C.-H.L.); day_7749@hotmail.com (J.-Y.C.); 3 Department of Biological Science and Technology, China Medical University, Taichung 40402, Taiwan; sllee@mail.cmu.edu.tw (S.-L.L.); mckao@mail.cmu.edu.tw (M.-C.K.); wawagay@hotmail.com (Y.-T.L.); 4 Graduate Institute of Integrated Medicine, China Medical University, Taichung 40402, Taiwan; cjchen@mail.cmu.edu.tw; 5 Proteomics Core Laboratory, Department of Medical Research, China Medical University Hospital, Taichung 40447, Taiwan; 6 Department of Biochemistry, National Defense Medical Center, Taipei 11466, Taiwan; 7 School of Pharmacy, China Medical University, Taichung 40402, Taiwan; 8 Graduate Institute of Natural Products, Kaohsiung Medical University, Kaohsiung 80708, Taiwan

**Keywords:** *Ipomoea batatas*, purple sweet potato leaves, glucose uptake effect

## Abstract

*Ipomoea batatas* has long been used in folk medicine for the treatment of hyperglycemia or as a food additive for the prevention of type 2 diabetes. However, neither the plant extract nor its active components have been evaluated systematically. In this work four crude extracts, including *n*-hexane- (IBH), 95% MeOH- (IBM), *n*-BuOH- (IBB), and H_2_O-soluble (IBW) fractions, were prepared by fractionation of a methanolic extract of purple *I. batatas* leaves. Twenty-four pure compounds **1**–**24** were then isolated by various chromatographic techniques and their structures identified from NMR and MS data. Glucose uptake assays in differentiated 3T3-L1 adipocytes and rat primary hepatocytes, as well as western blot analysis, were carried out to evaluate the antidiabetic activity of this species. The IBH crude fraction, with methyl decanoate (**22**) as a major and active compound, showed the greatest effect on glucose uptake, most likely via activation of Glut4 and regulation of the PI3K/AKT pathway. Quercetin 3-*O*-β-d-sophoroside (**1**), quercetin (**3**), benzyl β-d-glucoside (**10**), 4-hydroxy-3-methoxybenzaldehyde (**12**), and methyl decanoate (**22**) could be important components contributing to the antidiabetic effects. We conclude that purple *I. batatas* leaves have potential as an antidiabetic plant source and the active constituents **1**, **3**, **10**, **12**, and **22** are promising lead candidates for future investigation.

## 1. Introduction

*Ipomoea batatas* (L.) (Convolvulaceae), commonly known as sweet potato, is a well-known valuable medicinal food. While the roots and leaves of *I. batatas* play important roles as an energy source for humans and animals, they also have been used in traditional medicine for the treatment of various diseases [1]. Numerous pharmacological properties, including antidiabetic (caffeic acid derivatives, anthocyanosides, flavonoids, arabinogalactan-protein) [1,2,3], anti-oxidant (caffeic acid derivatives, anthocyanosides, coumarins) [1,4,5], anticancer (caffeic acid derivatives, anthocyanosides, coumarins) [1], antimicrobial (caffeic acid derivatives, triterpenes) [1], anticoagulant (coumarins) [1], and anti-inflammatory (resin glycosides) [6] activities, have been reported for this species. The green *I. batatas* leaves have long been used in folk medicine for the treatment of hyperglycemia or as a food additive for the prevention of type 2 diabetes [7,8,9], while the purple *I. batatas* leaves contain large quantities of rough fibers and in Taiwan they are usually discarded or fed to animals. A systematic investigation of the active antidiabetic compounds isolated from the latter variety has not been reported. Therefore, as part of our studies to identify promising antidiabetic drugs from natural products, the active components of purple sweet potato leaves were elucidated herein.

## 2. Results and Discussion

### 2.1. Glucose Uptake Efficacy of Four Crude Fractions Prepared from Purple I. batatas Leaves

A MeOH extract of the aerial parts of purple *I. batatas* was separated into *n*-hexane- (IBH), 95% MeOH- (IBM), *n*-BuOH- (IBB), and H_2_O-soluble (IBW) fractions by liquid-liquid partition chromatography (see Section 3.3. Extraction and Isolation). 3T3-L1 adipocyte and primary rat hepatocyte models were used to evaluate the glucose uptake efficacy of the above four fractions.

**Figure 1:**
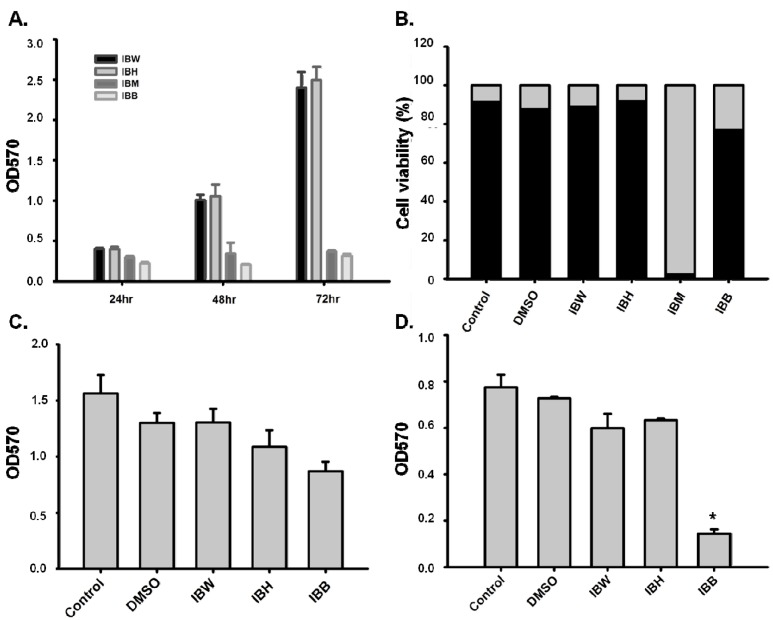
Cytotoxicity testing with IBH, IBM, IBB, and IBW crude fractions. (**A**) MTT assay for 3T3-L1 preadipocytes (1 × 10^4^ cells/cm^2^) treated with 0.1 mg/mL of IBH, IBM, IBB, and IBW; (**B**) Trypan blue assay for 3T3-L1 preadipocytes treated with extract (0.1 mg/mL) for 72 h. Survival and mortality rates of cells are marked in black and gray, respectively; (**C**) MTT assay of 3T3-L1 differentiated adipocytes (1 × 10^5^ cells/well, 12 wells) treated with 0.1 mg/mL of IBH, IBB, and IBW in 10% FBS medium for 72 h; (**D**) MTT assay of primary rat hepatocytes (1 × 10^5^ cells/well, 12 wells collagen coating) treated with 0.1 mg/mL of IBH, IBB, and IBW for 24 h. ANOVA statistical analysis, * *p <* 0.05.

**Figure 2:**
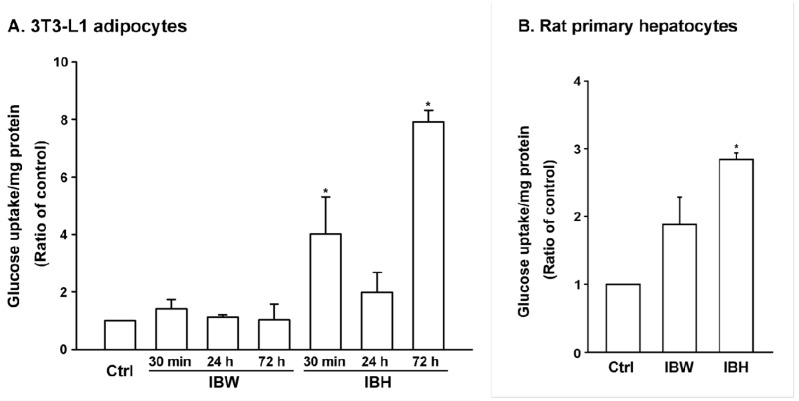
Glucose uptake test for IBH and IBW crude fractions. (**A**) Glucose uptake test in 3T3-L1 adipocytes: Differentiated 3T3-L1 adipocytes were treated with 0.1 mg/mL of IBW and IBH for 30 min, 24 h, and 72 h; (**B**) Glucose uptake test in rat primary hepatocytes: Hepatocytes (1 × 10^5^ cells/well, 12 wells collagen coating) were treated with 0.1 mg/mL of IBW and IBH for 24 h. The amount of 2-NBDG (2-[*N*-(7-nitrobenz-2-oxa-1,3-diazol-4-yl)amino]-2-deoxyglucose) taken up by cells was measured by the fluorescence at excitation and emission wavelengths (485 and 535 nm, respectively). DMSO (0.2%) was used as the control group for IBH. Statistical analysis was performed with ANOVA, * *p <* 0.05.

In MTT and trypan blue assays, IBM was cytotoxic to 3T3-L1 preadipocytes (Figure 1A,B). In contrast, IBH, IBB, and IBW exhibited no significant cytotoxicity toward differentiated 3T3-L1 preadipocytes in the MTT assay (Figure 1C), while IBB was cytotoxic to primary rat hepatocytes (Figure 1D). Therefore, only IBH and IBW were tested for glucose uptake in 3T3-L1 adipocytes and rat hepatocytes. IBH showed a significant effect in both models (Figure 2A,B), while IBW affected glucose uptake activity only in the latter model.

**Figure 3:**
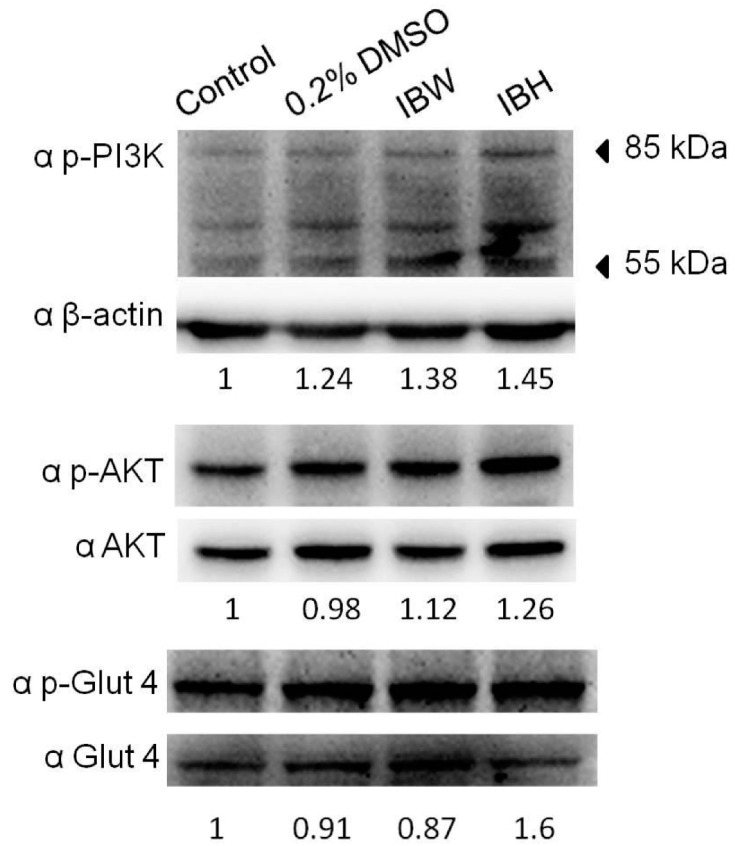
The effects of extracts on PI3K/AKT/Glut4 expression in mature 3T3-L1 adipocytes. Cells were treated with 0.1 mg/mL of IBW and IBH crude extracts for 24 h, and then analyzed for PI3K, AKT, Glut4, and β-actin by western blot analysis.

As shown in Figure 3, IBH increased the phosphorylation of PI3K, AKT, and Glut4 in 3T3-L1 adipocytes; however, IBW increased only p-PI3K. These data suggest that IBH might activate Glut4 by regulating the PI3K/AKT pathway.

### 2.2. Chemical Composition of the n-Hexane-Soluble Fraction (IBH)

The chemical composition of the *n*-hexane fraction (IBH) was analyzed by GC/MS as shown in Table 1 (see Supplementary Materials for details). The three major components in this fraction were esters of fatty and unsaturated carboxylic acids, specifically, methyl decanoate (**22**, 11.08%), methyl cinnamate (**23**, 14.12%), and methyl laurate (**24**, 14.24%). Among the 47 minor compounds also identified, terpenoids constituted 12.70%, including eleven sesquiterpenes (6.67%) and two monoterpenes (5.92%). Fourteen aromatic compounds (20.08%), two acids (1.16%), two additional esters (0.70%), three aldehydes (0.95%), two ketones (2.70%), two alcohols (1.27%), six alkanes (5.45%), and three alkenes (2.45%) were also determined (Table 1). However, other compounds (13.21%) in the IBH fraction remained unknown. An earlier report studied the volatiles from purple *I. batatas* leaves and identified major (germacrene D, germacrene B, caryophyllene, and *n*-hexadecanoic acid) and minor (benzaldehyde, nonanal, β-elemene, isocaryophyllene, (−)-β-caryophyllene epoxide *etc.*) constituents of this plant [10]. In the present study, we also found germacrene D (0.16%), benzaldehyde (0.22%), nonanal (0.50%), β-elemene (0.72%), isocaryophyllene (0.13%), and (−)-β-caryophyllene epoxide (2.69%) in *I. batatas*. However, the major components found in the two studies were not the same [10]. Different environment, plant cultivation, collection period, extraction methodology, and analytical techniques could account for the differences.

### 2.3. Chemical Composition of 95% MeOH- and n-BuOH-soluble Fractions (IBM and IBB)

**Figure 4:**
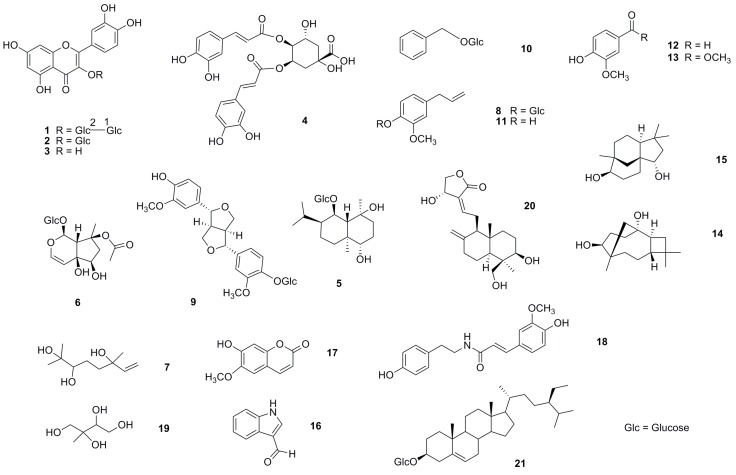
Structures of compounds **1**–**21** isolated from the IBM (compounds **10**–**21**) and IBB (compounds **1**–**9**) crude fractions.

Twenty-one components were isolated from the IBM (compounds **10**–**21**) and IBB (compounds **1**–**9**) extracts of purple *I. batatas* leaves (Figure 4 and Table S1). The polarities of compounds **1**–**21** in both crude fractions were higher than those in IBH. Three flavonoids, quercetin 3-*O*-β-d-sophoroside (**1**) [11], quercetin 3β-*O*-glucoside (**2**) [12], and quercetin (**3**) [12], one caffeic acid derivative, 3,4-di-*O*-caffeoyl isoquinic acid (**4**) [13], three sesquiterpenes, ananosmoside A (**5**) [14], caryolane-1,9β-diol (**14**) [15], and clovane-2β,9α-diol (**15**) [15], one iridoid glucoside, 8-*O*-acetyl-harpagide (**6**) [16], one monoterpenoid triol (**7**) [17,18], two phenylpropanoids, eugenyl *O*-β-d-glucopyranoside (**8**) [19] and eugenol (**11**) [20], one ligand, (+)-pinoresinol-β-d-glucoside (**9**) [21], three benzene derivatives, benzyl β-d-glucoside (**10**) [22], 4-hydroxy-3-methoxybenzaldehyde (**12**) [23], and methyl 4-hydroxy-3-methoxybenzoate (**13**) [24], one alkaloid, indole-3-aldehyde (**16**) [25], one coumarin, 6-methoxy-7-hydroxycoumarin (**17**) [26], one amide, *trans*-*N*-feruloyltyramine (**18**) [27], one isoprene derivative, 2-methyl-1,2,3,4-butanetetrol (**19**) [28], one diterpene, andrographolide (**20**) [29], and one steroid, sitosterol-3-β-d-glucose (**21**) [30] were identified from the NMR and MS spectroscopic data (see Supplementary Materials). Except for **3**, **17**, and **21**, the remaining compounds were reported for the first time from this plant.

### 2.4. Chemical Composition of the H_2_O-Soluble Fraction (IBW)

In the 1D and 2D NMR spectra (Figures S1–S6) of the IBW crude fraction, the major signals were similar to those of compound **19** (Figures S7 and S8), which was isolated from the IBM crude fraction. In the LC/MS analysis of IBW, peaks were found at *m*/*z* 137.0838 [M + H]^+^ and 135.0634 [M − H]^−^, which could possibly be assigned to compound **19**, although two different retention times were also observed (Figure S11). However, 2-methyl-1,2,3,4-butanetetrol (**19**) has four stereoisomers, (2*S*,3*R*)- or (2*R*,3*S*)-2-*C*-methylerythritol and (2*S*,3*S*)- or (2*R*,3*R*)-2-*C*-methylthreitol [28,31] (Figure S12). Thus, based on our data, the major constituent of IBW is 2-methyl-1,2,3,4-butanetetrol (**19**), occurring as a mixture of stereoisomers. Compound **19** is a photooxidative isoprene emitted by plants and has been reported as a major contributor to secondary organic aerosols in the atmosphere [31]. Besides, it could be speculated that saccharide components of the IBW crude fraction account for the remaining NMR chemical shifts between 3.0–6.5 and 60–110 ppm in the ^1^H- and ^13^C-NMR spectra, respectively (Figures S1 and S2). Acid hydrolysis and HPLC analysis possibility indicated d-galactose (15.94 min), l-galactose (16.53 min), d-glucose (18.34 min), l-arabinose (20.31 min), and d-arabinose (22.01 min) units in IBW extract compared with sugar derivatives standards: d-galactose (16.01 min), l-galactose (16.57 min), d-glucose (18.36 min), l-arabinose (20.51 min), and d-arabinose (21.85 min) (see Section 3.6 and Figure S13).

### 2.5. Glucose Uptake Effect of the Components from IBH, IBM, IBB, and IBW

**Figure 5:**
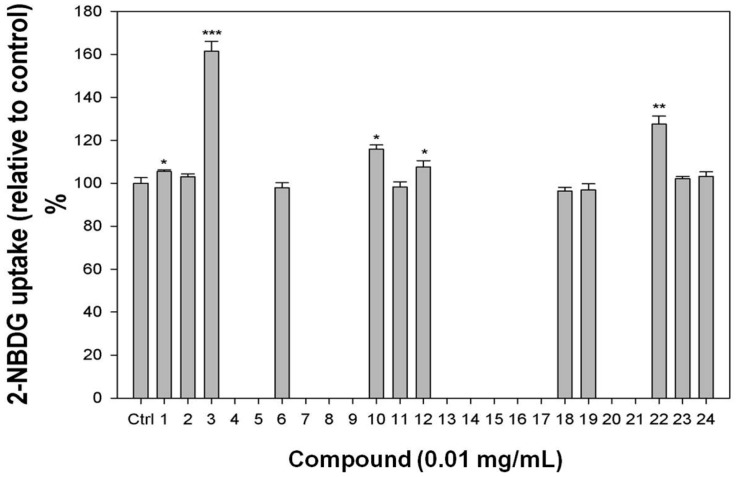
Glucose uptake test in differentiated 3T3-L1 adipocytes for pure compounds **1**–**24**. Differentiated 3T3-L1 adipocytes were treated with compound for 30 min, and then 2-NBDG uptake was measured. Compounds **4**–**5**, **7**–**9**, **13**–**17**, and **20**–**21** were cytotoxic toward 3T3-L1 adipocytes and were not tested in this assay. ANOVA statistical analysis: *, *p* < 0.05; **, *p* < 0.01; ***, *p* < 0.005.

Adipocytes regulation of glucose uptake can decrease postprandial hyperglycemia. To determine the effect of the isolates from purple *I. batatas* leaves on glucose uptake and identify potential promising antidiabetic natural products leads, we evaluated selected isolated compounds in an insulin-stimulated glucose uptake assay using differentiated 3T3-L1 adipocytes and a fluorescent d-glucose analog (2-NBDG). Because the IBH fraction showed significant effects on glucose uptake (Figure 2), its three major components **22**–**24** (Table 1), purchased from commercial companies (see Section 3.1. General Procedures), were studied. Among these three compounds, only methyl decanoate (**22**) increased glucose uptake (27.5%) relative to the control (Figure 5).

With the exception of **4**–**5**, **7**–**9**, **13**–**17**, and **20**–**21**, which were cytotoxic toward 3T3-L1 adipocytes and contributed to the cytotoxic properties of IBM and IBB, all components (Figure 4) isolated from IBM and IBB crude fractions were also tested in the glucose uptake assay. As shown in Figure 5, quercetin 3-*O*-β-d-sophoroside (**1**), quercetin (**3**), benzyl β-d-glucoside (**10**), and 4-hydroxy-3-methoxybenzaldehyde (**12**) increased 2-NBDG uptake rates by 5.4%, 61.4%, 15.8%, and 7.5%, respectively relative to the control. Compound **19**, which was found in IBM and also identified as the major component of IBW, did not affect the cells’ glucose uptake rate.

In people with type II diabetes, insulin-induced stimulation of hepatic, muscle, and adipose tissue glucose uptake is impaired [32,33,34,35]. Among numerous structurally diverse natural products affecting glucose uptake [35,36,37,38,39,40,41,42,43,44,45], flavonoids are a predominant factor [36,37,38,39,40]. Among isolates **1**–**24**, compounds **1**–**3** are flavonol derivatives and quercetin (**3**) was identified in our study as the most active single component of purple *I. batatas* leaves. In 2008, Fang reported that **3** could significantly improve insulin-stimulated glucose uptake in mature 3T3-L1 adipocytes by acting as a partial agonist of PPARγ [46]. Although flavonoid **1** is a sugar derivative of **3**, it had a much lower effect than **3** on glucose uptake. Additionally, compound **2** (flavonol-3-glucoside) did not affect glucose uptake, even though **2** is structurally similar to cyanidin-3-glucoside [39]. In 2011, Nidhina *et al.* reported that vanillin (4-hydroxy-3-methoxybenzaldehyde, **12**) could increase glucose uptake, as well as the expression levels of PPARγ and its target gene Glut4 in 3T3-L1 cells [47]. Our research study has first reported the glucose uptake activities of compounds **1**, **10**, and **22**. These compounds, as well as **3** and **12**, merit further research as possible natural products leads in antidiabetic drug development.

## 3. Materials and Methods 

### 3.1. General Procedures

1D and 2D NMR spectra were measured on a 500 MHz Avance III (Bruker, Rheinstetten, Germany). Chemical shift (δ) values are reported in ppm (part per million) with pyridine-*d*_5_ and methanol-*d*_4_ as internal standard, and coupling constants (*J*) are in Hz. Low-resolution ESIMS were measured on a Bruker Daltonics EsquireHCT ultra high capacity trap mass spectrometer. TLC was performed on Kieselgel 60 F_254_ (0.25 mm, Merck, Darmstadt, Germany) or RP-18 F_254_S (0.25 mm, Merck), and spots were viewed under UV light at 254 and 356 nm and then stained by spraying with 10% H_2_SO_4_ and heating on a hot plate. For column chromatography, silica gel (SILICYCLE 70–230 and 230–400 mesh), RP-18 (LiChroprep^®^ 40–63 μm, Merck), Sephadex™ LH-20 (GE Healthcare, Uppsala, Sweden) and Diaion^®^ HP-20 (Supelco™, Bellefonte, PA, USA) were used. A Shimadzu LC-20AT pump, Shimadzu RID-10A refractive index detector/SPD-M20A diode array detector (Shimadzu Inc., Kyoto, Japan) with a SUPELCO™ Ascentis^®^ and Discovery HS^®^ (250 × 10 mm i.d., 5 μm, C_18_) columns with 2 mL/min flow rate were used for HPLC. Methyl laurate (Chem Service, West Chester, PA, USA), methyl cinnamate (Aldrich, St. Louis, MO, USA), and methyl decanoate (Chem Service, West Chester, PA, USA) were used for GC/MS analysis.

### 3.2. Plant Material

Aerial parts of purple *I. batatas* leaves (5.0 kg) were collected in Luzhu District, Taoyuan City, Taiwan (121°17′7.28′′E, 25°4′42.03′′N), in July, 2013 by the author M.C. Kao. The plants were cultivated on Prof. Kao’s private farmland with his permission to conduct a study on this species. The plant was authenticated by the National Plant Resources Center of Taiwan Agricultural Research Institute with the account number Pin 375. A voucher specimen (IB2013-2014) was deposited at CMRDC, China Medical University Hospital, Taiwan.

### 3.3. Extraction and Isolation

The aerial parts of *I. batatas* (5.0 kg) were extracted three times with MeOH (30.0 L each) at room temperature to obtain a crude extract. The MeOH extract (350.0 g) was partitioned between EtOAc and H_2_O (1:1, *v*/*v*) to give an EtOAc-soluble fraction and an aqueous phase, which were further partitioned between *n*-hexane/95% MeOH and *n*-BuOH/H_2_O (1:1, *v*/*v*), respectively, to give n-hexane- (IBH), 95% MeOH- (IBM), n-BuOH- (IBB), and H_2_O-soluble (IBW) extracts.

Fractionation of extract IBM (30.0 g) was carried out by silica gel chromatography (column diameter: 9 cm, length: 20 cm; CH_2_Cl_2_/MeOH, 30:1), yielding six fractions (IBM1–IBM6). Fraction IBM1 (1.2 g) was subjected to open column chromatography on silica gel (column: 2.5 × 28 cm; CH_2_Cl_2_/*n*-hexane, 5:1) and gave eight subfractions (IBM1.1–IBM1.8). Subfraction IBM1.2 (58.7 mg) was partitioned by RP-18 chromatography (column: 2 × 27 cm; MeOH/H_2_O, 90:10) and purified by preparative TLC (*n*-hexane/EtOAc, 5:1) to give **11** (3.1 mg). Subfraction IBM1.5 (93.5 mg) was subjected to silica gel column chromatography (column: 2 × 30 cm; *n*-hexane/EtOAc, 5:1) to give pure **13** (11.1 mg). Subfraction IBM1.6 (67.0 mg) was partitioned by silica gel chromatography (column: 2 × 30 cm; *n*-hexane/EtOAc, 5:1) and purified by preparative TLC (CH_2_Cl_2_/MeOH, 5:1) to give **12** (3.8 mg). Fraction IBM2 (8.3 g) was divided into four subfractions by Sephadex LH-20 (column: 5 × 50 cm; CH_2_Cl_2_/MeOH, 1:1) and subfraction IBM2.3 (1.2 g) was separated into seven subfractions by column chromatography on silica gel (column: 2.5 × 28 cm; *n*-hexane/EtOAc, 3:1). Subfraction IBM2.3.6 (82.5 mg) was subjected to RP-18 column chromatography (column: 2 × 28 cm; MeOH/H_2_O, 3:2) and purified by preparative TLC (CH_2_Cl_2_/MeOH, 100:1) to give **17** (4.0 mg). Subfraction IBM2.4 (354.2 mg) was chromatographed on a silica gel column (column: 2.5 × 28 cm), using *n*-hexane/EtOAc (3:1) as eluent. Subfraction IBM2.4.5 (17.5 mg) was partitioned by silica chromatography (column: 1.5 × 28 cm; CH_2_Cl_2_/MeOH, 50:1) to give **16** (2.1 mg). Sephadex LH-20 chromatography (column: 5 × 50 cm; CH_2_Cl_2_/MeOH, 1:1) of Fraction IBM3 (9.0 g) gave compound **21** (100.0 mg) as well as four impure subfractions. Subfraction IBM3.3 (3.1 g) was subjected to RP-18 (column: 2.5 × 28 cm; MeOH/H_2_O, 3:1) chromatography to give IBM3.3.2 (1.3 g) and IBM3.3.4 (113.8 mg). Subfraction IBM3.3.2 was separated by RP-18 (column: 5 × 28 cm; MeOH/H_2_O, 1:1) into seven subfractions (IBM3.3.2.1–IBM3.3.2.7). Subfraction IBM3.3.2.4 (160.9 mg) was chromatographed on silica gel (column: 2 × 28 cm; EtOAc/MeOH, 100:1 and CH_2_Cl_2_/MeOH, 20:1), and further purified by preparative TLC (*n*-hexane/EtOAc, 1:1 and *n*-hexane/acetone, 1:1) to give **18** (3.4 mg). Subfraction IBM3.3.2.5 (177.1 mg) was subjected to silica gel (column: 2 × 28 cm; CH_2_Cl_2_/MeOH, 15:1) chromatography to give IBM3.3.2.5.2 (48.6 mg). The subfration IBM3.3.2.5.2 was purified by silica gel chromatography (column: 2 × 28 cm; *n*-hexane/EtOAc, 5:1 and *n*-hexane/acetone, 2:1) to give **20** (18.3 mg). Subfraction IBM3.3.4 was subjected to silica gel chromatography (column: 2 × 28 cm; EtOAc/MeOH, 100:1 and CH_2_Cl_2_/MeOH, 20:1) and purified by RP-HPLC (MeOH/H_2_O, 85:15) to give compounds **14** (4.9 mg, t_R_ = 11 min) and **15** (2.4 mg, t_R_ = 13 min). Fraction IBM5 (3.6 g) was fractionated into six subfractions by Sephadex LH-20 (column: 5 × 47 cm; MeOH). Subfraction IBM5.3 (476.7 mg) was subjected to silica gel chromatography (column: 2.5 × 28 cm; CH_2_Cl_2_/MeOH, 4:1 and column: 2.5 × 20 cm; CH_2_Cl_2_/MeOH, 3:1) to give **19** (9.0 mg).

The IBB extract (53.0 g) was chromatographed over Diaion^®^ HP-20 (column: 7.5 × 42 cm; H_2_O/MeOH/acetone, 100:0:0; 50:50:0; 0:100:0; 0:0:100) to give four fractions (IBB1–IBB4). Fraction IBB2 (14.3 g) was subjected to Sephadex LH-20 (column: 5 × 56 cm; MeOH) to obtain eight subfractions. Subfraction IBB2.3 (2.9 g) was separated by RP-18 chromatography (column: 2.5 × 28 cm; MeOH/H_2_O, 1:2) to give six subfractions (IBB2.3.1–IBB2.3.6), and subfraction IBB2.3.4 (620.5 mg) was further subjected to RP-18 chromatography (column: 2.5 × 28 cm; MeOH/H_2_O, 1:1.5) to give three subfractions (IBB2.3.4.1–IBB2.3.4.3). Subfraction IBB2.3.4.2 (531.9 mg) was separated into three subfractions (IBB2.3.4.2.1–IBB2.3.4.2.3) and IBB2.3.4.2.1 (45.5 mg) was purified by silica gel chromatography (column: 2 × 28 cm; CHCl_3_/MeOH, 6:1) to give **7** (5.6 mg). Subfraction IBB2.3.4.2.2 (305.9 mg) was subjected to RP-18 chromatography (column: 2 × 28 cm; MeOH/H_2_O, 1:1.5 and 1:2) and silica gel chromatography (column: 2 × 28 cm; EtOAc/MeOH, 50:1) to give **6** (12.7 mg). Subfraction IBB2.3.5 (997.0 mg) was separated by silica gel chromatography (column: 2 × 28 cm; CHCl_3_/MeOH, 8:1, EtOAc/MeOH, 20:1) and RP-18 chromatography (column: 2 × 28 cm; MeOH/H_2_O, 1:2.5) to obtain **5** (18.4 mg). Subfraction IBB2.4 (2.0 g) was partitioned by RP-18 chromatography (column: 2 × 28 cm; MeOH/H_2_O, 1:2) and its subfraction IBB2.4.2 (436.5 mg) was isolated by RP-18 chromatography (column: 2 × 28 cm; MeOH/H_2_O, 1:2.5 and 1:2) and then purified by preparative TLC (CH_2_Cl_2_/MeOH, 8:1) to give **10** (4.4 mg). Subfraction IBB2.5 (3.3 g) was separated into six subfractions by column chromatography with RP-18 gel (column: 2 × 28 cm; MeOH/H_2_O, 1:1.5), and subfraction IBB2.5.2 (2.1 g) was subjected to RP-18 chromatography (column: 2 × 28 cm; MeOH/H_2_O, 1:1) to give **1** (613.7 mg). Subfraction IBB2.5.3 (972.6 mg) was purified on silica gel (column: 2 × 28 cm; EtOAc/MeOH, 3:1) and then was subjected to RP-18 chromatography (column: 2 × 28 cm; MeOH/H_2_O, 1:1, 1:1.5) to give **2** (20.6 mg). Subfraction IBB2.6 (652.2 mg) was subjected to RP-18 chromatography (column: 2 × 28 cm; MeOH/H_2_O, 1:1, 1:2) to give **4** (39.9 mg). Fraction IBB3 (3.9 g) yielded nine subfractions (IBB3.1–IBB3.9) after Sephadex LH-20 (column: 5 × 48 cm, MeOH) chromatography. The subfraction IBB3.3 (170.0 mg) was subjected to open column chromatography on silica gel (column: 2 × 25 cm, CHCl_3_/MeOH, 7:1) and further purified by RP-HPLC (MeCN/H_2_O, 20:80) to give **8** (8.0 mg, t_R_ = 16 min). Subfraction IBB3.4 (220.0 mg) was separated by silica gel chromatography (column: 2 × 25 cm; CHCl_3_/MeOH, 7:1) and RP-18 chromatography (column: 1.5 × 21.5 cm; MeOH/H_2_O, 40:60) to obtain **9** (7.0 mg). Subfraction IBB3.9 (52.0 mg) was purified by RP-HPLC (MeOH/H_2_O, 50:50) to give **3** (2.0 mg, t_R_ = 32 min).

### 3.4. GC/MS Analysis of the n-Hexane-soluble Fraction (IBH)

(1) HS-SPME analysis: A 50/30-μm divinylbenzene/carboxen/polydimethylsiloxane fiber (Supelco, Inc.) was used for aroma extraction. IBH samples was put into a 7 mL vial (Hole Cap PTFE/Silicone Septa) and sealed. The SPME fiber was exposed to each sample for 30 min at 25 °C, after which each sample was injected into a gas chromatograph injection unit [48].
(2) Analysis of the components of samples by GC and GC/MS: Qualitative and quantitative analyses of the volatile compounds were conducted using a 7890A GC (Agilent, Wilmington, DE, USA) equipped with a 60 m × 0.25 mm i.d. DB-1 fused-silica capillary column with a film thickness of 0.25 μm and a flame ionization detector. The injector and detector temperatures were maintained at 250 °C and 300 °C, respectively. The oven temperature was held at 40 °C for 1 min and then raised to 150 °C at 5 °C/min and held for 1 min, finally raised to 200 °C at 10 °C/min and held for 11 min. The carrier gas (nitrogen) flow rate was 1 mL/min. Kovats indices were calculated for the separated components relative to a C_5_-C_25_ *n*-alkane mixture [49]. Percentage composition was calculated using the peak area normalization measurements.
(3) Analysis of the components of the samples by GC-MS: The volatile compounds were identified using an Agilent 7890B GC equipped with a 60 m × 0.25 mm i.d. DB-1 fused-silica capillary column with a film thickness of 0.25 μm coupled to an Agilent model 5977 A MSD mass spectrometer (MS). The injector temperature was maintained at 250 °C. The GC conditions in the GC-MS analysis were the same as in the GC analysis. The carrier gas (helium) flow rate was 1 mL/min. The electron energy was 70 eV at 230 °C. The constituents were identified by matching their spectra with those recorded in a MS library (Wiley 7n) [48].

### 3.5. LC/MS Analysis of H_2_O-Soluble Fraction (IBW)

A UHPLC system (Ultimate 3000; Dionex, Germering, Germany) equipped with a C18 reversed-phase column (2.1 × 150 mm, 3 μm, T3; Waters, Milford, MA, USA) was coupled with a hybrid Q-TOF mass spectrometer (maXis impact, Bruker Daltonics, Bremen, Germany) with an orthogonal electrospray ionization (ESI) source. The initial flow rate was 0.25 mL/min of 99% solvent A (0.1% formic acid) and 1% solvent B (MeCN with 0.1% formic acid). A volume of 2 μL of sample was injected. After injection, solvent B was maintained at 1% for 4 min, then increased to 45% during a span of 14 min, and finally to 99% over a period of 2 min after which this percentage composition was held for 2 min. After 0.5 min, solvent B was reduced back to 1% and held at this percentage for 2.5 min.

The mass spectrometer was operated in either positive or negative ion mode using the *m*/*z* range 50–1000 at 1 Hz (summation value of 9839). The capillary voltage of the ion source and negative was set at +4500 V for positive mode and −2500 V for negative mode, and the endplate offset was 500 V. The nebulizer gas flow was 1 bar and drying gas flow was 8 L/min. The drying temperature was set at 200 °C. Funnel 1 radiofrequency (RF) and Funnel 2 RF were both 200 Vpp. The hexapole RF was 30 Vpp and the low mass cutoff of quadrupole was 100 *m*/*z*. For the MS/MS settings, the eight most intense ions from each MS full scan spectrum were automatically selected as the precursor ion peaks for the following auto MS/MS experiments.

### 3.6. Acid Hydrolysis and Reversed-Phase HPLC Analysis of IBW

The IBW crude fraction (10.0 mg) was hydrolyzed in 1 M HCl/1,4-dioxane (1:1, 2.0 mL) at 90 °C for 3 h and then partitioned with CH_2_Cl_2_/H_2_O (1:1). The aqueous layer was neutralized with Amberlite IRA400. After drying, the residue was dissolved in pyridine (1.0 mL) containing L-cysteine methyl ester hydrochloride (10.0 mg) and heated at 60 °C for 1 h. A 10 μL solution of *o*-tosyl isothiocyanate in pyridine was added to the mixture, which was heated at 60 °C for 1 h. The final reaction mixture was directly analyzed by HPLC: Analytical HPLC was performed on a 250 × 4.6 mm i.d. Cosmosil 5C18-AR II column at 35 °C with isocratic elution of 25% CH_3_CN in 50 mM H_3_PO_4_ for 40 min at a flow rate 0.8 mL/min [50].

### 3.7. Cell Culture

The 3T3-L1 preadipocytes were cultured as previously described [51]. The cells were grown in Dulbecco’s modified Eagle’s medium (DMEM) with high glucose containing 10% (*v*/*v*) FBS, 100 U/mL penicillin and 100 μg/mL streptomycin in plates (10^5^ cells/mL) at 37 °C in a humidified atmosphere of 10% CO_2_. The medium was changed every two days. Primary rat hepatocytes (HCs) were isolated and cultured as previously described [52]. Primary HCs were suspended in William’s Medium E containing 10% (*v*/*v*) FBS, 100 U/mL penicillin, 100 μg/mL streptomycin, 2 mM L-glutamine, 0.86 μM insulin, 0.5 nM dexamethasone, and 10 mM HEPES and were plated on collagen-coated dishes (5 × 10^5^ cells/mL). Cells were cultured at 37 °C with 5% CO_2_ for 3 h for attachment and were washed twice with PBS. The medium was then changed, and, the cells were used for experiments after overnight incubation.

### 3.8. Cell Viability and Proliferation

The cells were treated with different concentrations of crude fractions or pure compounds for the indicated time points. Cell viability was assayed using the Trypan blue exclusion method. The cells were stained with Trypan blue. The unstained (viable) and stained (dead) cells were counted separately using a hemocytometer under a microscope (Nikon TS100). The cell viability was calculated based on the ratio of viable cells to total cell population in each plate. Cell proliferation was assayed using MTT metabolic analysis. MTT was added to the cell medium, and after incubation at 37 °C for 4 h, the blue formazan reduction product was dissolved in isopropanol and measured on an ELISA reader at 570 nm [51].

### 3.9. Differentiation of 3T3-L1 Cells into Mature Adipocytes

3T3-L1 cells were cultured from preadipocytes and differentiated into adipocytes as previously described. Preadipocytes were grown in Dulbecco’s modified Eagle’s medium (DMEM) with high glucose containing 10% (*v*/*v*) FBS, 100 U/mL penicillin, and 100 μg/mL streptomycin in plates (10^5^ cells/mL) at 37 °C in a humidified atmosphere of 10% CO_2_. The medium was changed every 2 days. Then, two days after confluence, the medium was replaced with DMEM (high glucose) containing 10% FBS and adipogenic agents (1.7 μM insulin, 0.25 μM DMX, and 0.5 mM IBMX); this day was designated day 0 and occurred after three days of culturing. The cells were then grown in DMEM containing 10% FBS, and the medium was changed every two days. Differentiated cells were used for experimentation on day 9 when the proportion of differentiated cells reached up to 90%. The differentiated cells were identified by Oil Red O staining and by gradient centrifugation with Percoll [53].

### 3.10. Western Blot Analysis

The cells were treated as indicated, detached, thoroughly washed with PBS, and then lysed in ice-cold lysis buffer. Following centrifugation at 13,000× *g* for 10 min at 4 °C, the supernatants (30 μg protein) were boiled with reducing sample buffer for 5 min, subjected to electrophoresis in SDS-polyacrylamide gels, and then transferred onto a PVDF membrane. The membrane was blocked with 1% BSA in PBS containing 0.1% Tween-20 (PBST) for 1 h at room temperature and then washed with PBST. Proteins were detected by incubating the membrane overnight at 4 °C with antibodies against β-actin (Sigma-Aldrich), Glut4 (IF8), p-Glut4 (Ser 488) (Santa Cruz Biotechnology, Santa Cruz, CA, USA), Akt (pan), p-Akt (Ser 473), p-PI3K (Tyr458/Tyr199) (Cell Signaling Technology, Danvers, MA, USA). Next, the membrane was washed with PBST, and, finally, the membrane was incubated with a secondary antibody conjugated to horseradish peroxidase (HRP) for 1 h. An enhanced chemiluminescence (ECL) kit (Amersham Biosciences, Arlington Heights, IL, USA or Millipore, Billerica, MA, USA) was used for protein detection.

### 3.11. Glucose Uptake Assay

The differentiated 3T3-L1 adipocytes or primary rat hepatocytes were cultured with low glucose DMEM for 6 h, and then PBS including 1% bovine serum albumin (BSA) was added. The crude extracts (0.1 mg/mL) and pure compounds (0.01 mg/mL) were added to the medium for the indicated time and then stimulated with 10^−7^ M insulin at 37 °C for another 30 min. A 10^−4^ M fluorescent d-glucose analog (2-[*N*-(7-nitrobenz-2-oxa-1,3-diazol-4-yl)amino]-2-deoxyglucose, 2-NBDG) was added to the former 3T3-L1 adipocytes for 30 min and then PBS was used to wash the cells three times. Some cell lysis buffers were used to obtain the cell extracts, which were homogenized with an ultrasonicator to assay the fluorescence with excitation and emission at 485 and 535 nm, respectively.

## 4. Conclusions 

In conclusion, four crude fractions (IBH, IBM, IBB, and IBM) were prepared from purple *I. batatas* leaves and 21 compounds were isolated. The IBH crude extract, with methyl decanoate (**22**) as a major and active component, showed antihyperglycemic potential *in vitro*. In a glucose uptake assay, flavonoids **1** and **3**, as well as benzene derivatives **10** and **12**, were identified as promising lead compounds, especially quercetin (**3**). Overall, our data systematically demonstrated that purple sweet potato leaves are a potential antidiabetic plant source. We believe that further investigation is definitely merited.

## Figures and Tables

**Table 1:** Chemical composition of *n*-hexane-soluble fraction from purple *I. batatas* leaves.

*R*_t_ (min) ^a^	RI ^b^	Compound	Peak Area (%)	Classification
12.04	860	Pentanoic acid	0.37	acid
14.21	927	Benzaldehyde	0.22	aldehyde
15.11	955	Hexanoic acid	0.79	acid
15.25	959	6-Methyl-5-hepten-2-one	0.68	ketone
15.89	978	1,2,4-Trimethylbenzene	0.61	aromatic compound
16.44	994	Decane	0.37	alkane
16.77	1004	1,2,3-Trimethylbenzene	1.23	aromatic compound
16.86	1007	2-Ethylhexanol	0.96	alcohol
17.64	1033	*m*-Diethylbenzene	0.29	aromatic compound
17.72	1036	*m*-Propyltoluene	0.49	aromatic compound
17.93	1043	*o*-Cymene	1.30	aromatic compound
18.21	1052	*o*-Propyltoluene	0.31	aromatic compound
18.53	1062	2-Ethyl-1,4-dimethylbenzene	1.06	aromatic compound
18.59	1063	2-Ethyl-1,3-dimethylbenzene	2.25	aromatic compound
18.70	1067	3-Methyldecane	0.20	alkane
18.87	1072	6-Methyl-3,5-heptadien-2-one	2.02	ketone
19.06	1078	Nonanal	0.50	aldehyde
19.41	1088	4-Ethyl-1,2-dimethylbenzene	0.83	aromatic compound
19.59	1094	Undecane	2.59	alkane
19.79	1100	1,2,3,4-Tetramethylbenzene	3.63	aromatic compound
19.90	1104	1,2,4,5-Tetramethylbenzene	4.38	aromatic compound
20.39	1121	(+)-2-Bornanone	1.82	monoterpene
20.54	1127	1,3-Diethyl-5-methylbenzene	0.58	aromatic compound
20.73	1133	l-Menthone	4.10	monoterpene
20.86	1138	1-Ethyl-3,5-dimethylbenzene	1.52	aromatic compound
21.46	1159	Butoxyethoxyethanol	0.31	alcohol
21.62	1104	Naphthalene	1.60	aromatic compound
22.15	1182	Decanal	0.23	aldehyde
22.26	1186	1-Dodecene	1.45	alkene
22.59	1196	Dodecane	1.71	alkane
25.20	1289	Methyl 4-decenoate	0.59	ester
25.48	1297	Tridecane	0.15	alkane
25.66	1303	Methyl decanoate	11.08	ester
26.85	1356	Methyl cinnamate	14.12	ester
27.46	1382	Bicyclogermacrene	0.54	sesquiterpene
27.52	1384	α-Copaene	0.94	sesquiterpene
27.62	1388	*trans*-2-Tetradecene	0.23	alkene
27.74	1393	β-Elemene	0.72	sesquiterpene
27.85	1398	Tetradecane	0.43	alkane
27.98	1404	Methyl undecanoate	0.11	ester
28.06	1408	(−)-Isoledene	0.18	sesquiterpene
28.46	1429	Isocaryophyllene	0.13	sesquiterpene
28.61	1437	β-Cubebene	0.12	sesquiterpene
29.25	1470	Aromadendren	0.38	sesquiterpene
29.42	1479	Germacrene D	0.16	sesquiterpene
29.71	1493	β-Selinene	0.81	sesquiterpene
29.96	1506	Methyl laurate	14.24	ester
31.38	1578	1*Z*,5*E*-7-Dodecatriene	0.77	alkene
31.53	1586	(-)-β-Caryophyllene epoxide	2.69	sesquiterpene
		Total	86.79	

^a^ *R*_t_: Retention time (min); ^b^ RI: Retention index, using paraffin (C_5_–C_25_) as reference.

## Supplementary Materials

Supplementary materials can be accessed at: http://www.mdpi.com/1420-3049/21/6/745/s1.

## Abbreviations

The following abbreviations are used in this manuscript:

TLC: Thin-Layer Chromatography
RP-18: Reversed-Phase Carbon-18
EtOAc: Ethyl Acetate
*n*-BuOH: Normal Butanol
MeOH: Methanol
HPLC: High-Performance Liquid Chromatography
NMR: Nuclear Magnetic Resonance
HSQC: Heteronuclear Single Quantum Coherence
HMBC: Heteronuclear Multiple Bond Correlation
COSY: Correlation Spectroscopy
NOESY: Nneclear Overhauser Exchange Spectroscopy
ESIMS: Electrospray Ionisation Mass Spectrometry
GC/MS: Gas Chromatography-Mass
LC/MS: Liquid Chromatography-Mass
*t*_R_: Retention Time

## References

[1] Mohanraj R., Sivasankar S. (2014). Sweet potato (*Ipomoea batatas* [L.] Lam)—A valuable medicinal food: A review. J. Med. Food..

[2] Li F., Li Q., Gao D., Peng Y. (2009). The optimal extraction parameters and anti-diabetic activity of flavonoids from *Ipomoea batatas* leaf. Afr. J. Trad. CAM.

[3] Oki N., Nonaka S., Ozaki S. (2011). The effects of an arabinogalactan-protein from the white-skinned sweet potato (*Ipomoea batatas* L.) on blood glucose in spontaneous diabetic mice. Biosci. Biotechnol. Biochem..

[4] Zhao J.G., Yan Q.Q., Xue R.Y., Zhang J., Zhang Y.Q. (2014). Isolation and identification of colourless caffeoyl compounds in purple sweet potato by HPLC-DAD-ESI/MS and their antioxidant activities. Food Chem..

[5] Dini I., Tenore G.C., Dini A. (2006). New polyphenol derivative in *Ipomoea batatas* tubers and its antioxidant activity. J. Agric. Food Chem..

[6] Yoshikawa K., Yagi C., Hama H., Tanaka M., Arihara S., Hashimoto T. (2010). Ipomotaosides A–D, resin glycosides from the aerial parts of *Ipomoea batatas* and their inhibitory activity against COX-1 and COX-2. J. Nat. Prod..

[7] Ludvik B., Waldhäusl R.P., Kautzky-Willer A., Pacini G. (2003). Mode of action of *Ipomoea batatas* (Caiapo) in type 2 diabetic patients. Metabolism.

[8] Ludvik B., Neuffer B., Pacini G. (2004). Efficacy of *Ipomoea batatas* (Caiapo) on diabetes control in type 2 diabetic subjects treated with diet. Diabetes Care.

[9] Ludvik B., Hanefeld M., Pacini G. (2008). Improved metabolic control by *Ipomoea batatas* (Caiapo) is associated with increased adiponectin and decreased fibrinogen levels in type 2 diabetic subjects. Diabetes Obes. Metab..

[10] Wang M., Xiong Y., Zeng M., Li H., Zhang T., Liang Y. (2010). GC-MS combined with chemometrics for analysis of the components of the essential oils of sweet potato leaves. Chromatographia.

[11] Cui E.J., Song N.Y., Shrestha S., Chung I.S., Kim J.Y., Jeong T.S., Baek N.I. (2012). Flavonoid glycosides from cowpea seeds (*Vigna sinensis* K.) inhibit LDL oxidation. Food Sci. Biotechnol..

[12] Fossen T., Pedersen A.T., Andersen Ø.M. (1998). Flavonoids from red onion (*Allium cepa*). Phytochemistry.

[13] Ono M., Masuoka C., Odake Y., Ikegashira S., Ito Y., Nohara T. (2000). Antioxidative constituents from *Tessaria integrifolia*. Food Sci. Technol. Res..

[14] Xu X., Xie H., Hao J., Jiang Y., Wei X. (2010). Eudesmane sesquiterpene glucosides from lychee seed and their cytotoxic activity. Food Chem..

[15] Liu H.Y., He H.P., Gao S., Chen C.X., Shen Y.M., Hao X.J. (2006). Two new diterpenoids from *Callicarpa pedunculata*. Helv. Chim. Acta..

[16] Morvai M., Nagy T., Kocsis Á., Szabó L.F., Podányi B. (2000). Effect of oxygen substituents on two- and three-bond carbon-proton spin-spin coupling constants. Magn. Reson. Chem..

[17] Ishikawa T., Kondo K., Kitajima J. (2003). Water-soluble constituents of coriander. Chem. Pharm. Bull..

[18] Williams P.J., Strauss C.R., Wilson B. (1980). New linalool derivatives in muscat of *Alexandria* grapes and wines. Phytochemistry.

[19] Takeda Y., Ooiso Y., Masuda T., Honda G., Otsuka H., Sezik E., Yesilada E. (1998). Iridoid and eugenol glycosides from *Nepeta cadmea*. Phytochemistry.

[20] Elgendy E.M., Khayyat S.A. (2008). Oxidation reactions of some natural volatile aromatic compounds: Anethole and eugenol. Russ. J. Org. Chem..

[21] Kim M.R., Moon H.T., Lee D.G., Woo E.R. (2007). A new lignan glycoside from the stem bark of *Styrax japonica* S. et Z. Arch. Pharm. Res..

[22] Seigler D.S., Pauli G.F., Nahrstedt A., Leen R. (2002). Cyanogenic allosides and glucosides from *Passiflora edulis* and *Carica papaya*. Phytochemistry.

[23] Ito J., Chang F.R., Wang H.K., Park Y.K., Ikegaki M., Kilgore N., Lee K.H. (2001). Anti-AIDS agents. 48. Anti-HIV activity of moronic acid derivatives and the new melliferone-related triterpenoid isolated from Brazilian propolis. J. Nat. Prod..

[24] Yin X.J., Xu G.H., Sun X., Peng Y., Ji X., Jiang K., Li F. (2010). Synthesis of bosutinib from 3-methoxy-4-hydroxybenzoic acid. Molecules.

[25] Nakajima E., Nakano H., Yamada K., Shigemori H. (2002). Isolation and identification of lateral bud growth inhibitor, indole-3-aldehyde, involved in apical dominance of pea seedlings. Phytochemistry.

[26] Jerezano A., Jiménez F., Cruz M.C., Montiel L.E., Delgado F., Tamariz J. (2011). New approach for the construction of the coumarin frame and application in the total synthesis of natural products. Helv. Chim. Acta.

[27] Yamazaki Y., Kawano Y., Uebaysai M. (2008). Induction of adiponectin by natural and synthetic phenolamides in mouse and human preadipocytes and its enhancement by docosahexaenoic acid. Life Sci..

[28] Ghosh S.K., Butler M.S., Lear M.J. (2012). Synthesis of 2-*C*-methylerythritols and 2-*C*-methylthreitols via enantiodivergent sharpless dihydroxylation of trisubstituted olefins. Tetrahedron Lett..

[29] Matsuda T., Kuroyanagi M., Sugiyama S., Umehara K., Ueno A., Nishi K. (1994). Cell differentiation-inducing diterpenes from *Andrographis paniculata*. Chem. Pharm. Bull..

[30] Faizi S., Ali M., Saleem R., Irfanullah, Bibi S. (2001). Complete ^1^H- and ^13^C-NMR assignments of stigma-5-en-3-*O*-β-glucoside and its acetyl derivative. Magn. Reson. Chem..

[31] Nozière B., González N.J.D., Borg-Karlson A.K., Pei Y., Redeby J.P., Krejci R., Dommen J., Prevot A.S.H., Anthonsen T. (2011). Atmospheric chemistry in stereo: A new look at secondary organic aerosols from isoprene. Geophys. Res. Lett..

[32] Basu A., Basu R., Shah P., Vella A., Johnson C.M., Nair K.S., Jensen M.D., Schwenk W.F., Rizza R.A. (2000). Effects of type 2 diabetes on the ability of insulin and glucose to regulate splanchnic and muscle glucose metabolism: Evidence for a defect in hepatic glucokinase activity. Diabetes.

[33] Iozzo P., Hallsten K., Oikonen V., Virtanen K.A., Kemppainen J., Solin O., Ferrannini E., Knuuti J., Nuutila P. (2003). Insulin-mediated hepatic glucose uptake is impaired in type 2 diabetes: Evidence for a relationship with glycemic control. J. Clin. Endocrinol. Metab..

[34] Arha D., Pandeti S., Mishra A., Srivastava S.P., Srivastava A.K., Narender T., Tamrakar A.K. (2015). Deoxyandrographolide promotes glucose uptake through glucose transporter-4 translocation to plasma membrane in L6 myotubes and exerts antihyperglycemic effect *in vivo*. Eur. J. Pharmacol..

[35] Gao Y., Zhang M., Wu T., Xu M., Cai H., Zhang Z. (2015). Effects of D-pinitol on insulin resistance through the PI3K/Akt signaling pathway in type 2 diabetes mellitus rats. J. Agric. Food Chem..

[36] Lee H., Li H., Noh M., Ryu J.H. (2016). Bavachin from *Psoralea corylifolia* improves insulin-dependent glucose uptake through insulin signaling and AMPK activation in 3T3-L1 adipocytes. Int. J. Mol. Sci..

[37] Lee W., Yoon G., Kim M.C., Kwon H.C., Bae G.U., Kim Y.K., Kim S.N. (2016). 5,7-Dihydoxy-6-geranylflavanone improves insulin sensitivity through PPARα/γ dual activation. Int. J. Mol. Sci..

[38] Krishna M.S., Joy B., Sundaresan A. (2015). Effect on oxidative stress, glucose uptake level and lipid droplet content by apigenin 7,4′-dimethyl ether isolated from *Piper longum* L.. J. Food Sci. Technol..

[39] Matsukawa T., Inaquma T., Han J., Villareal M.O., Isoda H. (2015). Cyanidin-3-glucoside derived from black soybeans ameliorate type 2 diabetes through the induction of differentiation of preadipocytes into smaller and insulin-sensitive adipocytes. J. Nutr. Biochem..

[40] Nquyen P.H., Ji D.J., Han Y.R., Choi J.S., Rhyu D.Y., Min B.S., Woo M.H. (2015). Selaginellin and biflavonoids as protein tyrosine phosphatase 1B inhibitors from *Selaginella tamariscina* and their glucose uptake stimulatory effects. Bioorg. Med. Chem..

[41] Thakkar C.S., Kate A.S., Desai D.C., Ghosh A.R., Kulkarni-Almeida A.A. (2015). NFAT-133 increase glucose uptake in L6 myotubes by activating AMPK pathway. Eur. J. Pharmacol..

[42] Kato E., Inaqaki Y., Kawabata J. (2015). Higenamine 4′-*O*-β-d-glucoside in the lotus plumule induces glucose uptake of L6 cells through β2-adrenergic receptor. Bioorg. Med. Chem..

[43] Shyni G.L., Kavitha S., Indu S., Arya A.D., Anusree S.S., Vineetha V.P., Vandana S., Sundaresan A., Raghu K.G. (2014). Chebulagic acid from *Terminalia chebula* enhances insulin mediated glucose uptake in 3T3-L1 adipocytes via PPARγ signaling pathway. Biofactors..

[44] Kotowska D., EI-Houri R.B., Borkowski K., Petersen R.K., Frette X.C., Wolber G., Grevsen K., Christensen K.B., Christensen L.P., Kristiansen K. (2014). Isomeric C12-alkamides from the roots of *Echinacea purpurea* improve basal and insulin-dependent glucose uptake in 3T3-L1 adipocytes. Planta Med..

[45] Cai J., Zhao L., Zhu E., Guo J. (2014). Stimulating effect of a new triterpene derived from *Anoectochilus elwesii* on glucose uptake in insulin-resistant human HepG2 cells. Nat. Prod. Res..

[46] Fang X.K., Gao J., Zhu D.N. (2008). Kaempferol and quercetin isolated from *Euonymus alatus* improve glucose uptake of 3T3-L1 cells without adipogenesis activity. Life Sci..

[47] Nidhina P.A.H., Poulose N., Gopalakrishnapillai A. (2011). Vanillin induces adipocyte differentiation in 3T3-L1 cells by activating extracellular signal regulated kinase 42/44. Life Sci..

[48] Yeh C.H., Tsai W.Y., Chiang H.M., Wu C.S., Lee Y.I., Lin L.Y., Chen H.C. (2014). Headspace solid-phase microextraction analysis of volatile components in *Phalaenopsis* Nobby’s Pacific Sunset. Molecules.

[49] Schomburg G., Dielmann G. (1973). Identification by means of retention parameters. J. Chromatogr. Sci..

[50] Tanaka T., Nakashima T., Ueda T., Tomii K., Kouno I. (2007). Facile discrimination of aldose enantiomers by reversed-phase HPLC. Chem. Pharm. Bull..

[51] Lee S.L., Lee H.K., Chin T.Y., Tu S.C., Kuo M.H., Kao M.C., Wu Y.C. (2015). Inhibitory effects of purple sweet potato leaf extract on the proliferation and lipogenesis of the 3T3-L1 preadipocytes. Am. J. Chin. Med..

[52] Lee S.L., Chin T.Y., Lai C.L., Wang W.H. (2015). *Sedum mexicanum* Britt. induces apoptosis of primary rat activated hepatic stellate cells. Evid. -Based Complement. Altern. Med..

[53] Lee S.L., Chin T.Y., Tu S.C., Wang Y.J., Hsu Y.T., Kao M.C., Wu Y.C. (2015). Purple sweet potato leaf extract induces apoptosis and reduces inflammatory adipokine. Evid. Based Complement. Altern. Med..

