# Supplementary material for: Characterization of Secondary Metabolites from Purple Ipomoea batatas Leaves and Their Effects on Glucose Uptake
Source: Molecules. 2016 Jun 8;21(6):745. doi: 10.3390/molecules21060745 (PMC6273172; doi:10.3390/molecules21060745)
Supplement: Supplementary file 1 [file molecules-21-00745-s001.pdf]

Supplementary Materials: Characterization of Secondary Metabolites from Purple *Ipomoea batatas* Leaves and Their Effects on Glucose Uptake

Chia-Lin Lee, Shou-Lun Lee, Chao-Jung Chen, Hsin-Chun Chen, Ming-Ching Kao, Chuan-Hao Liu, Jau-Yang Chen, Yen-Ting Lai and Yang-Chang Wu

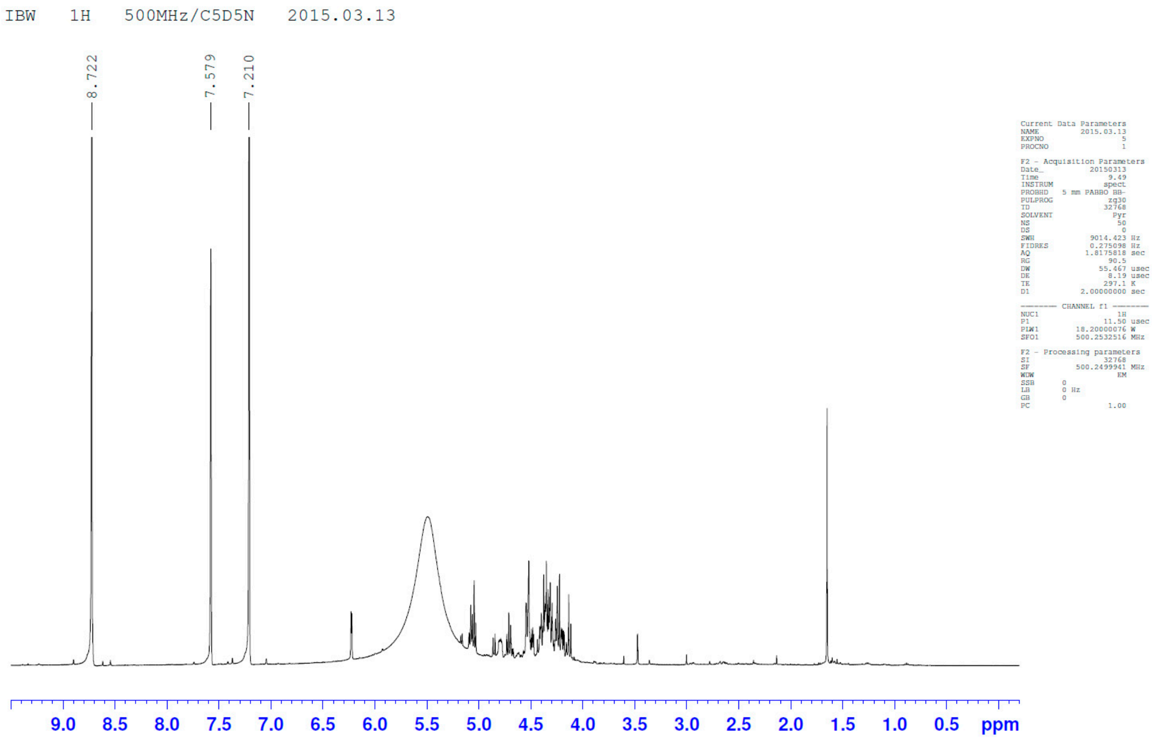

Figure S1. ^{1}H-NMR (500 MHz, C_5D_5N) of IBW.

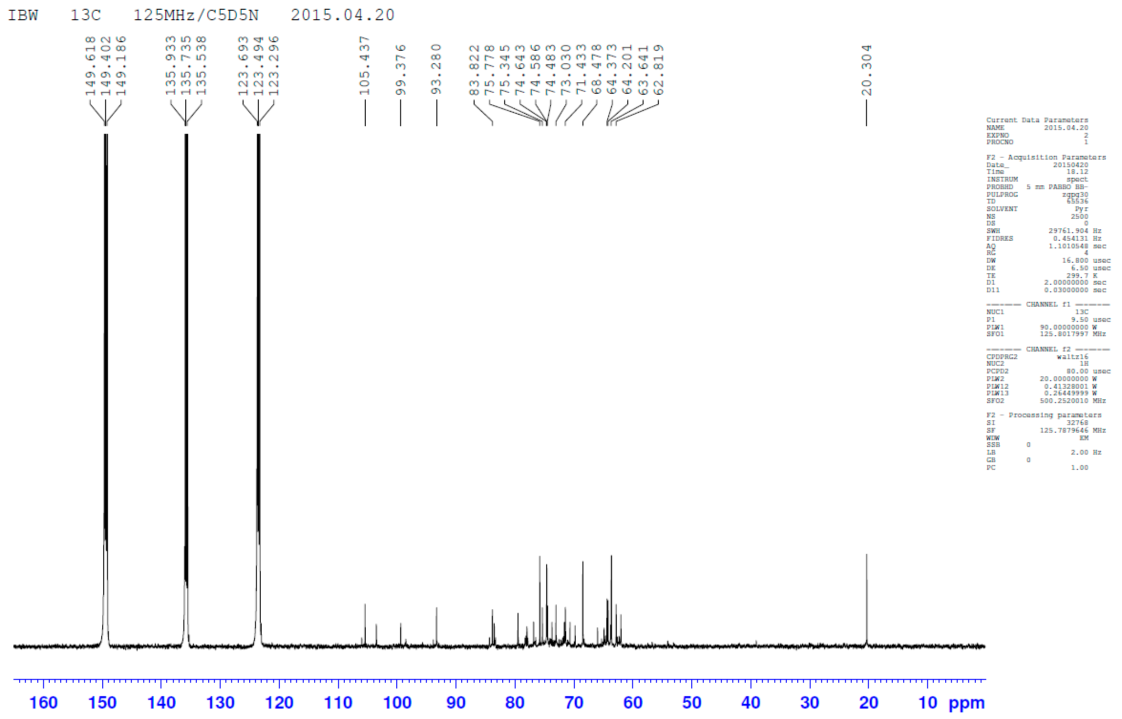

Figure S2. ^{13}C-NMR (125 MHz, C_5D_5N) of IBW.

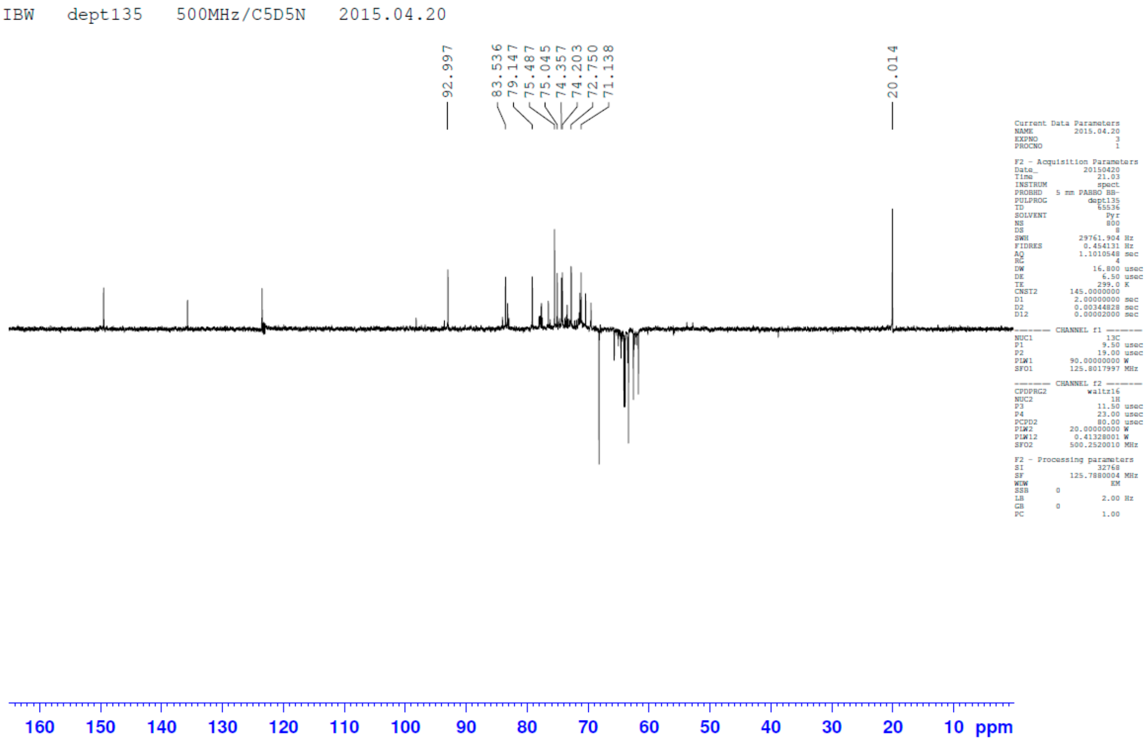

Figure S3. DEPT135 NMR (500 MHz, C_5D_5N) of IBW.

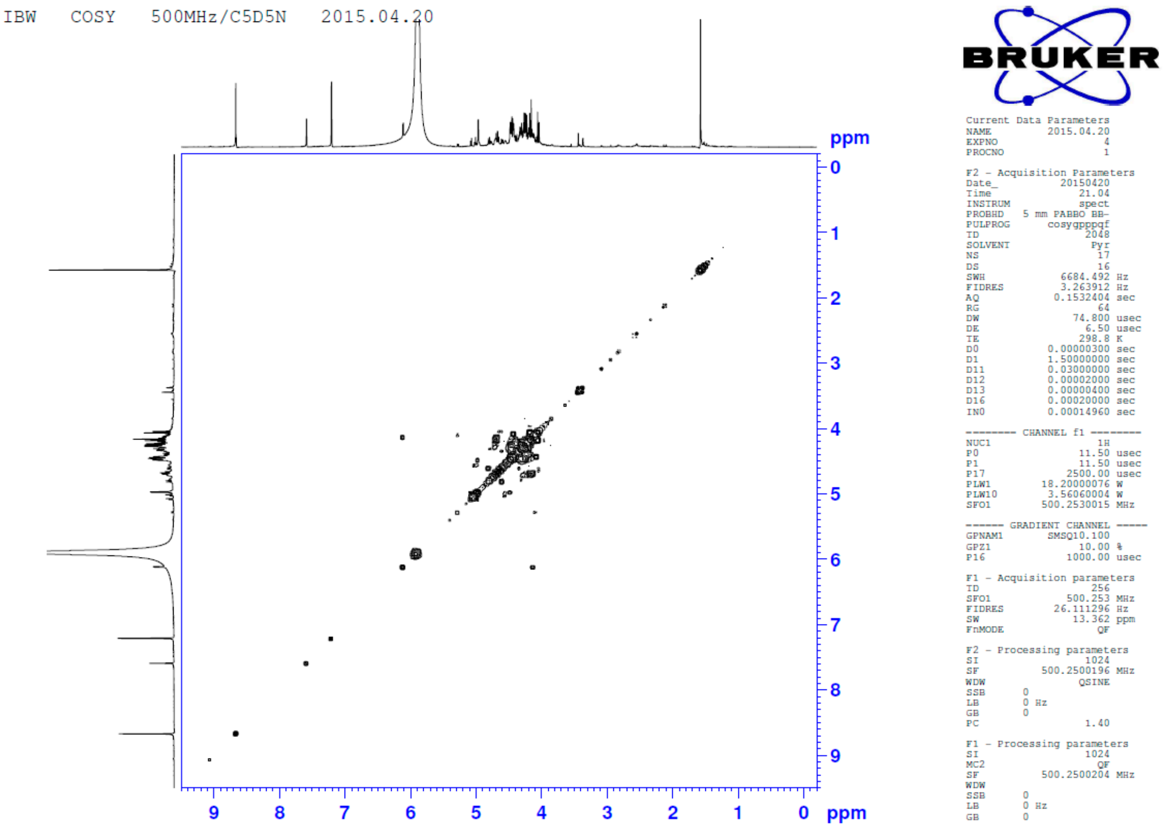

Figure S4. COSY NMR (500 MHz, C_5D_5N) of IBW.

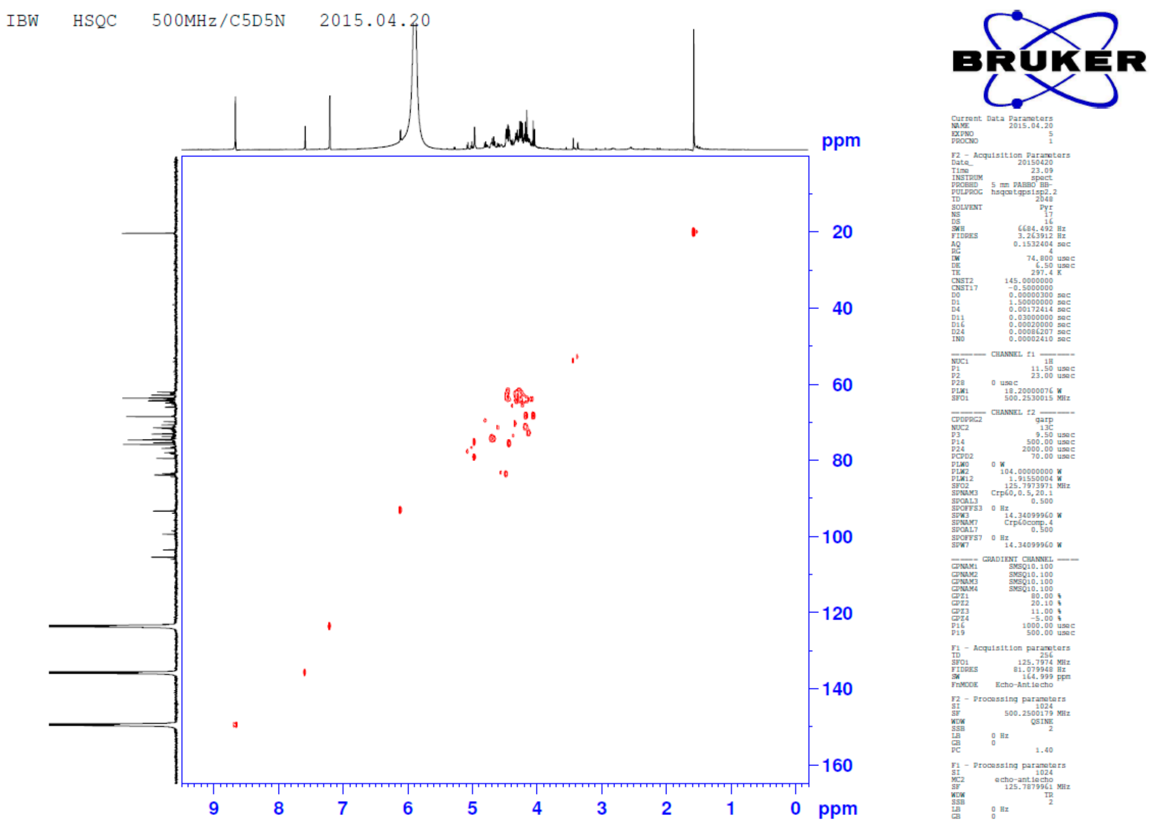

Figure S5. HSQC NMR (500 MHz, C_5D_5N) of IBW.

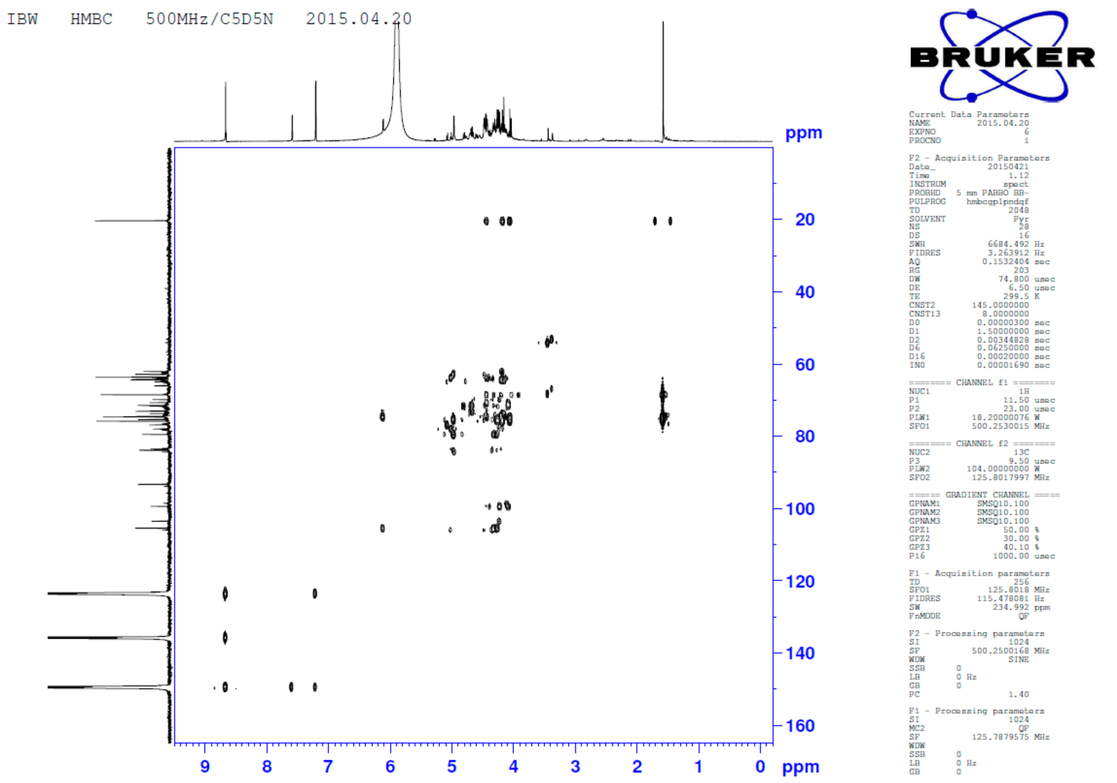

Figure S6. HMBC NMR (500 MHz, C_5D_5N) of IBW.

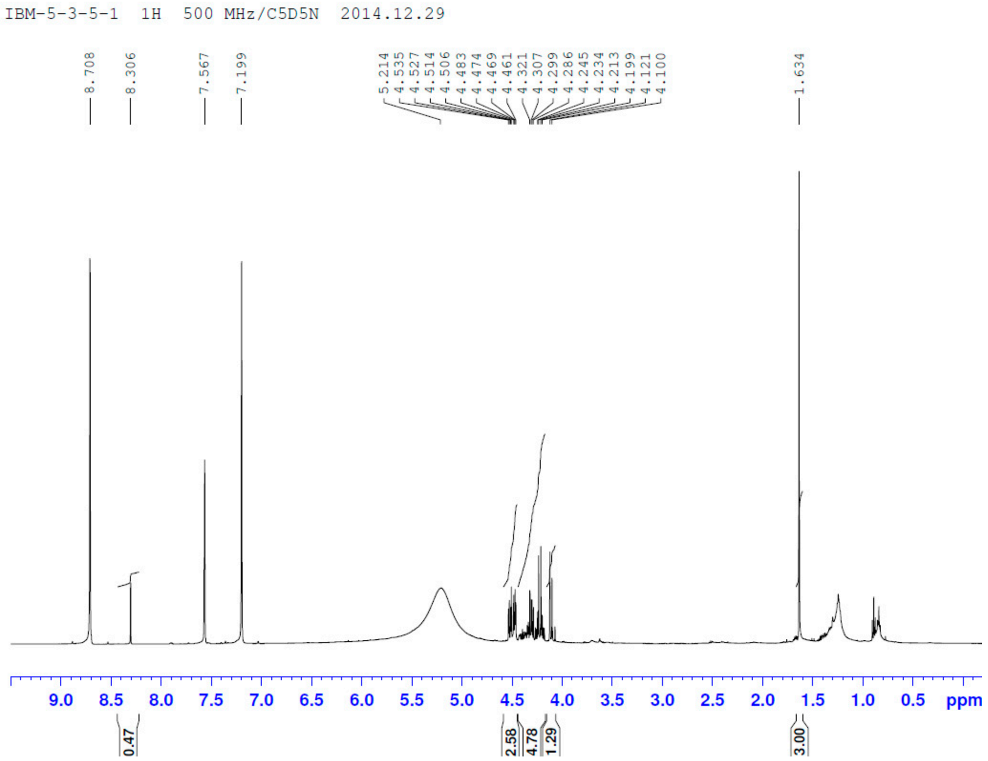

Figure S7. ^{1}H-NMR (500 MHz, C_5D_5N) of compound 19 isolated from IBM.

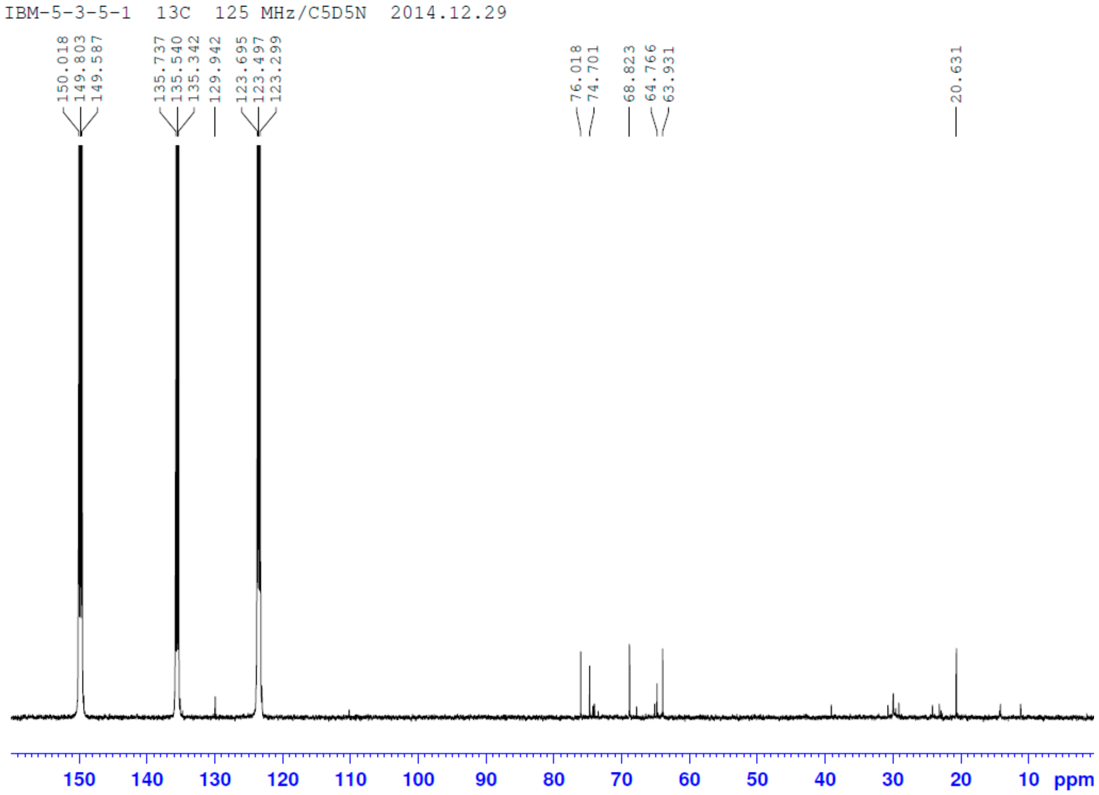

Figure S8. ^{13}C-NMR (125 MHz, C_5D_5N) of compound 19 isolated from IBM.

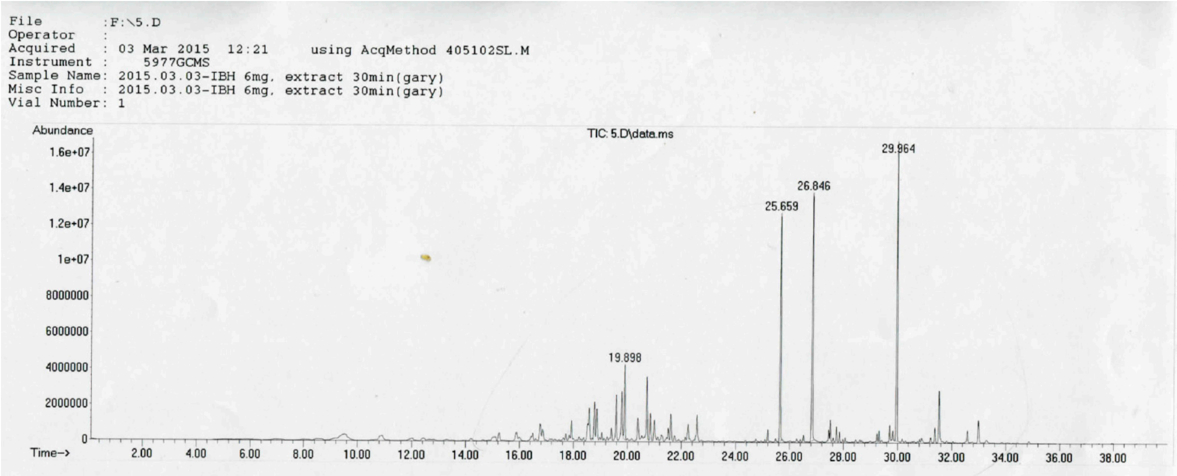

Figure S9. GC/MS analysis of *n*-hexane-soluble fraction (IBH).

Three major compounds (39.44%)

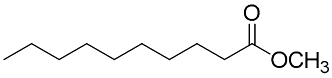
Methyl decanoate (11.08%)

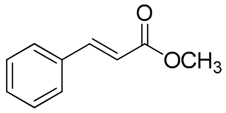
Methyl cinnamate (14.12%)

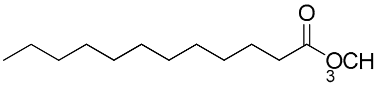
Methyl laurate (14.24%)

Figure S10. Three major compounds of IBH fraction.

Sesquiterpenes (6.67%)

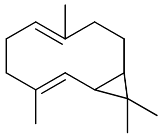
Bicyclogermacrene (0.54%)

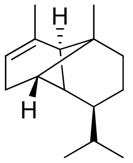
alpha-Copaene (0.94%)

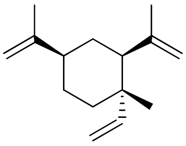
beta-Elemene (0.72%)

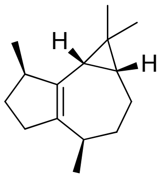
(-)-Isoledene (0.18%)

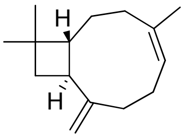
Isocaryophyllene (0.13%)

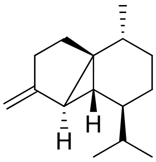
beta-Cubeben (0.12%)

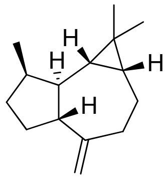
Aromadendrene (0.38%)

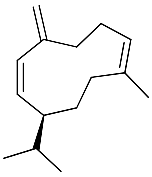
Germacrene D (0.16%)

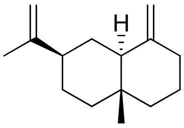
beta-Selinene (0.81%)

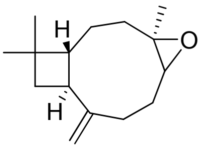
(-)-beta-Caryophyllene oxide (2.69%)

Figure S11. Sesquiterpenes of IBH fraction.

Monoterpenes (5.92%)

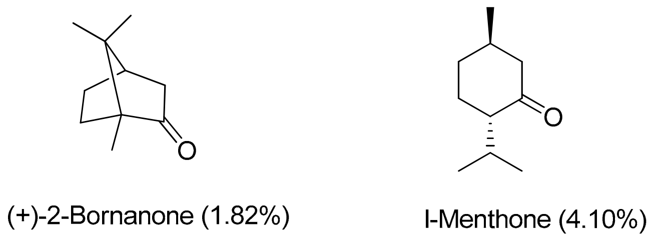

Figure S12. Monoterpenes of IBH fraction.

Aromatic compounds (20.08%)

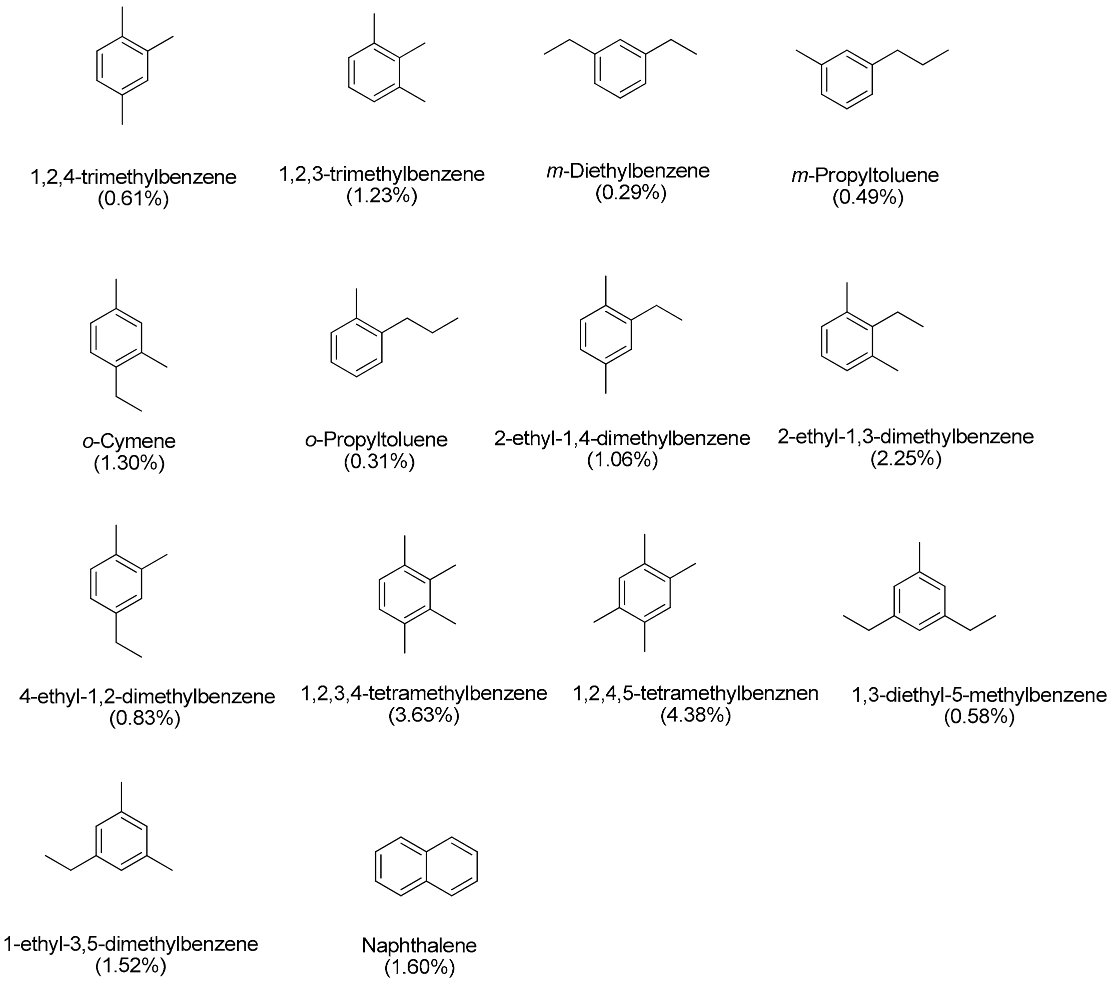

Figure S13. Aromatic compounds of IBH fraction.

Acids (1.16%), esters (0.70%), aldehydes (0.95%), ketones (2.70%), alcohols (1.27%)

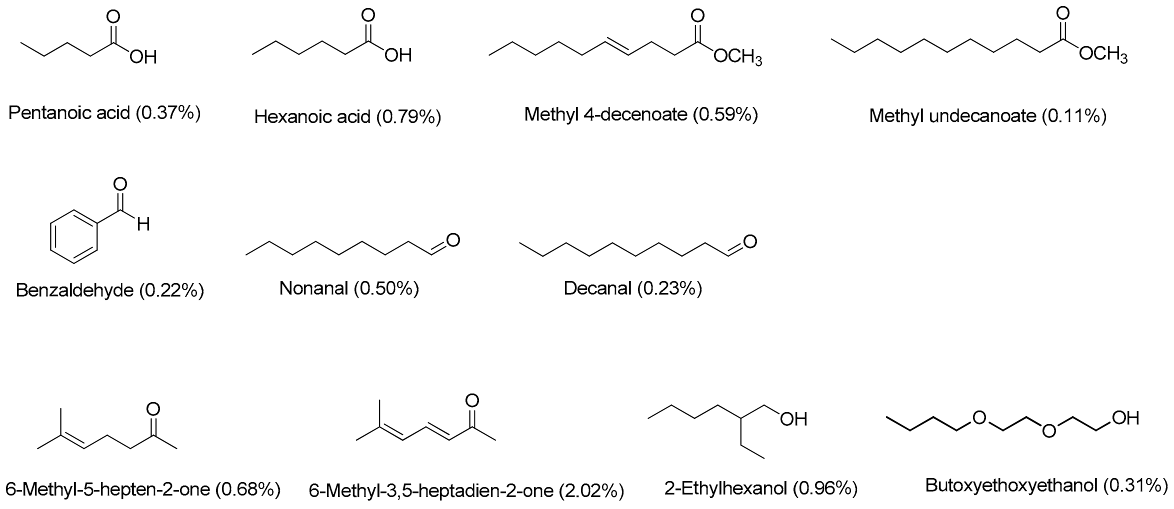

Figure S14. Acids, esters, aldehydes, ketones, and alcohols of IBH fraction.

Alkanes (5.45%), alkenes (2.45%)

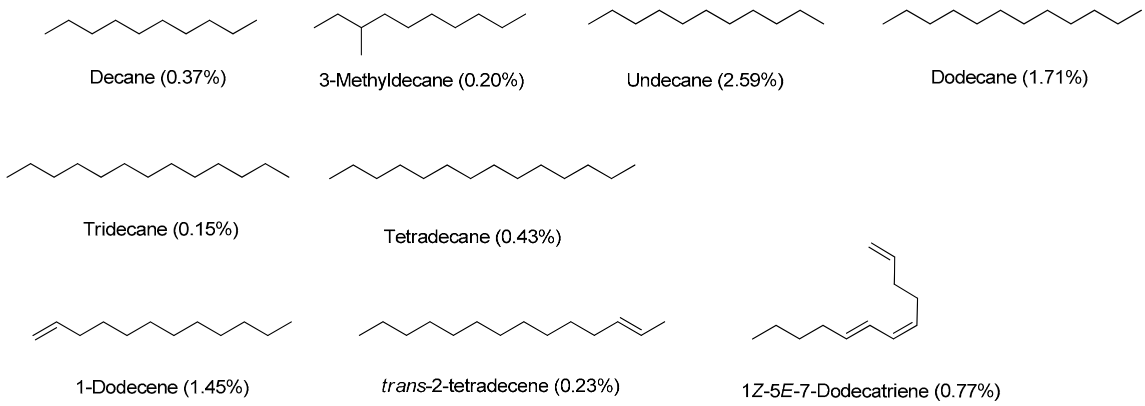

Figure S15. Alkanes and alkenes of IBH fraction.

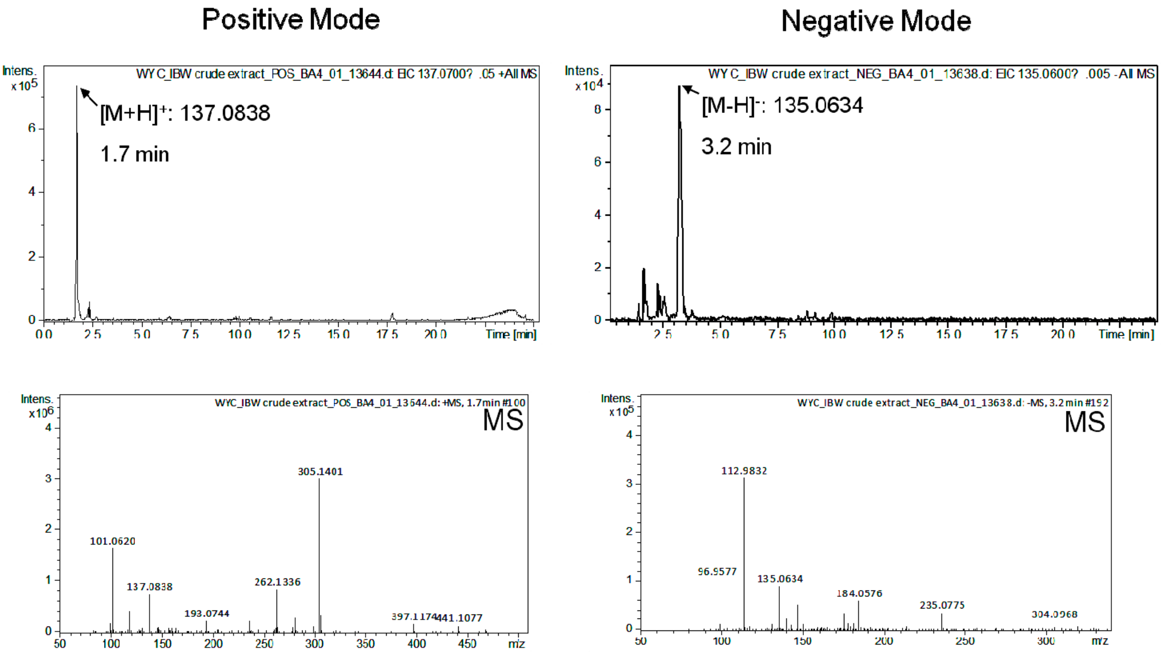

Figure S16. LC/MS analysis of H_2O-soluble fraction (IBW).

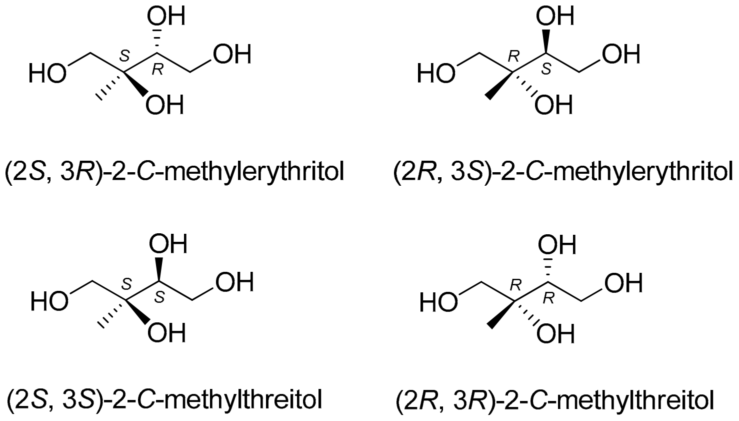

Figure S17. Four stereoisomers of 2-methyl-1,2,3,4-butanetetrol.

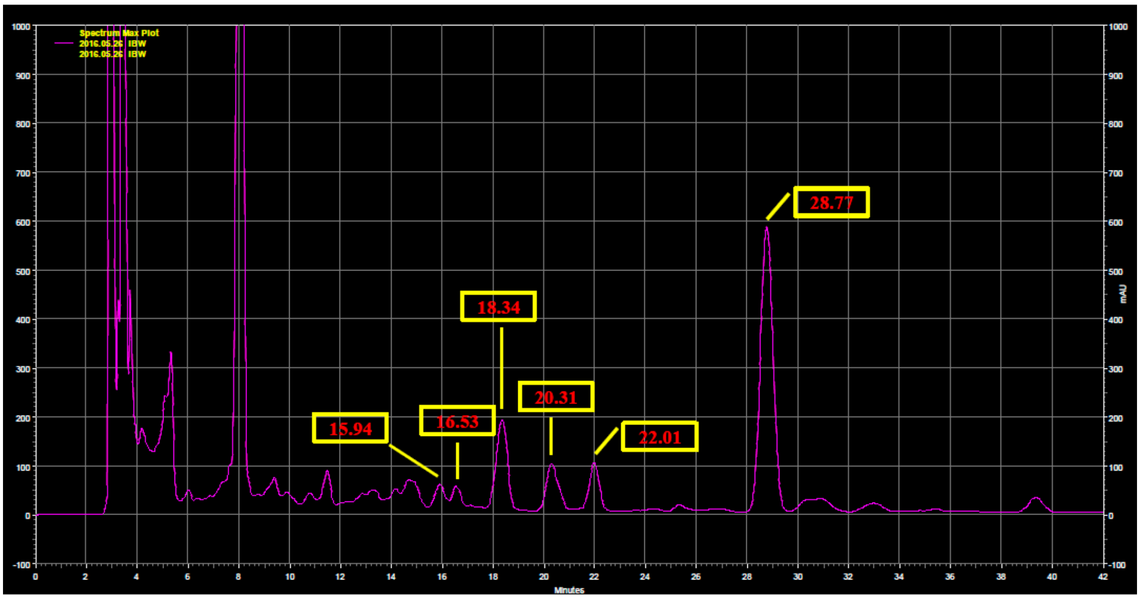

Figure S18. HPLC sugar derivatives analysis of IBW.

Table S1. Chemical composition of IBM and IBB crude fractions from purple *I. batatas* leaves.

IBM Crude Fraction	IBB Crude Fraction
benzyl β-D-glucoside (**10**)	quercetin 3-*O*-β-D-sophoroside (**1**)
eugenol (**11**)	quercetin 3β-*O*-glucoside (**2**)
4-hydroxy-3-methoxybenzaldehyde (**12**)	quercetin (**3**)
methyl 4-hydroxy-3-methoxybenzoate (**13**)	3,4-di-*O*-caffeoyl isoquinic acid (**4**)
caryolane-1,9β-diol (**14**)	ananosmoside A (**5**)
clovane-2β,9α-diol (**15**)	8-*O*-acetyl-harpagide (**6**)
indole-3-aldehyde (**16**)	triol (**7**)
6-methoxy-7-hydroxycoumarin (**17**)	eugenyl *O*-β-D-glucopyranoside (**8**)
trans-*N*-feruloyltyramine (**18**)	(+)-pinoresinol-β-D-glucoside (**9**)
2-methyl-1,2,3,4-butanetetrol (**19**)	
andrographolide (**20**)	
sitosterol-3-β-D-glucose (**21**)	

Appendix S1: Characterization of all compounds isolated from IBM and IBB fractions

Compound **1**: ^{1}H-NMR (C_5D_5N, 500 MHz): 3.94 (m), 4.10–4.49 (7H), 4.57 (dd, *J* = 12.0, 2.5 Hz), 5.63 (d, *J* = 7.5 Hz), 6.54 (d, *J* = 2.0 Hz), 6.62 (d, *J* = 7.5 Hz), 6.67 (d, *J* = 2.0 Hz), 7.38 (d, *J* = 8.5 Hz), 8.03 (dd, *J* = 8.5, 2.0 Hz), 8.40 (d, *J* = 2.0 Hz), 13.49 (brs); ^{13}C-NMR (C_5D_5N, 125 MHz): 62.1, 62.3, 70.9, 71.2, 76.1, 78.3, 78.4 (2C), 78.8, 84.5, 94.2, 99.5, 99.9, 105.1, 106.3, 116.3, 117.8, 122.4, 122.6, 134.5, 146.5, 150.4, 156.6, 157.2, 162.8, 165.4, 178.8; ESIMS *m*/*z* 625.0 $[M - H]^-$.

Compound **2**: ^{1}H-NMR (C_5D_5N, 500 MHz): 4.19–4.44 (6H), 6.21 (d, *J* = 7.5 Hz), 6.64 (d, *J* = 2.0 Hz), 6.69 (d, *J* = 2.0 Hz), 7.27 (d, *J* = 8.5 Hz), 8.06 (dd, *J* = 8.5, 2.0 Hz), 8.46 (d, *J* = 2.0 Hz), 13.17 (brs); ^{13}C-NMR (C_5D_5N, 125 MHz): 62.6, 71.4, 76.1, 78.6, 78.9, 94.5, 99.8, 104.5, 105.2, 116.2, 117.9, 122.3, 122.6, 134.5, 146.7, 149.8, 157.6, 157.7, 162.7, 165.9, 178.7; ESIMS *m*/*z* 487.1 $[M + Na]^+$, 462.9 $[M - H]^-$.

Compound **3**: ^{1}H-NMR (C_5D_5N, 500 MHz): 6.73 (d, *J* = 2.0 Hz), 6.77 (d, *J* = 2.0 Hz), 7.40 (d, *J* = 8.5 Hz), 8.12 (dd, *J* = 8.5, 2.0 Hz), 8.63 (d, *J* = 2.5 Hz), 13.34 (brs); ESIMS *m*/*z* 303.1 $[M + H]^+$, 300.9 $[M - H]^-$.

Compound **4**: ^{1}H-NMR (C_5D_5N, 500 MHz): 2.61–2.75 (4H), 4.58 (brs), 6.09 (brs), 6.49 (d, *J* = 10.5 Hz), 6.57 (d, *J* = 16.0 Hz), 6.70 (d, *J* = 16.0 Hz), 7.03 (d, *J* = 8.0 Hz), 7.10 (d, *J* = 8.0 Hz), 7.14 (d, *J* = 8.5 Hz), 7.21 (overlap), 7.50 (brs), 7.60 (brs), 7.94 (d, *J* = 16.0 Hz), 8.01 (d, *J* = 16.0 Hz); ^{13}C-NMR (C_5D_5N, 125 MHz): 38.27 (2C), 67.83, 69.32, 73.59, 74.80, 114.97, 115.07, 115.67, 115.87, 116.59 (2C), 122.18, 122.24, 126.76 (2C), 146.10, 146.25, 147.47, 147.55, 150.33, 150.44, 166.99 (2C), 182.50; ESIMS *m*/*z* 517.4 $[M + H]^+$, 539.1 $[M + Na]^+$, 515.0 $[M - H]^-$.

Compound **5**: ^{1}H-NMR (C_5D_5N, 500 MHz): 0.99 (3H, d, *J* = 6.5 Hz), 1.23 (3H, s), 1.42 (3H, d, *J* = 6.0 Hz), 1.59, 1.73 (3H, s), 1.90 (2H), 1.97, 2.20, 2.33, 2.46, 3.66, 3.67, 3.94–4.37 (4H), 4.59 (brd, *J* = 11.0 Hz), 5.00 (overlap), 5.01; ^{13}C-NMR (C_5D_5N, 125 MHz): 14.5, 22.9, 23.2, 23.5, 24.4, 25.8, 29.1, 36.4, 40.8, 41.6, 42.3, 51.2, 63.0, 71.9, 72.3, 75.6, 78.4, 78.5, 78.8, 79.2, 100.3; ESIMS *m*/*z* 441.3 $[M + Na]^+$.

Compound **6**: ^{1}H-NMR (C_5D_5N, 500 MHz): 1.58 (3H, s), 1.83 (3H, s), 2.01 (dd, *J* =15.0, 4.5 Hz), 2.53 (d, *J* = 15.0 Hz), 3.51 (s), 3.96, 4.04, 4.06, 4.21, 4.25, 4.31 (dd, *J* = 12.0, 5.5 Hz), 4.50 (dd, *J* = 11.5, 2.0 Hz), 5.15 (overlap), 5.30 (d, *J* = 7.5 Hz), 6.51 (d, *J* = 6.0 Hz), 6.68 (s); ^{13}C-NMR (C_5D_5N, 125 MHz): 21.9, 22.5, 45.7, 55.2, 62.7, 71.6, 72.9, 74.7, 76.8, 78.3, 78.6, 87.2, 94.5, 99.2, 107.9, 142.2, 170.9; ESIMS *m*/*z* 429.1 $[M + Na]^+$, 405.0 $[M - H]^-$.

Compound **7**: ^{1}H-NMR (C_5D_5N, 500 MHz): 1.47 (3H, s), 1.49 (3H, s), 1.50 (3H, s), 1.92, 1.94, 2.20, 2.42, 3.77 (dd, *J* = 10.0, 1.5 Hz), 6.20 (dd, *J* = 17.0, 10.5 Hz), 5.14 (dd, *J* = 6.5, 2.0 Hz), 5.78 (dd, *J* = 17.5, 2.0 Hz); ^{13}C-NMR (C_5D_5N, 125 MHz): 25.9, 26.1, 26.9, 28.7, 41.1, 72.5, 72.7, 79.8, 111.1, 147.5; ESIMS *m*/*z* 211.2 $[M + Na]^+$, 399.2 $[2M + Na]^+$, 187.1 $[M - H]^-$.

Compound **8**: ^{1}H-NMR (C_5D_5N, 500 MHz): 3.32 (2H, d, *J* = 7.0 Hz), 3.73 (3H, s), 4.11, 4.34–4.40 (4H), 4.53 (brd, *J* = 11.0 Hz), 5.08 (2H), 5.66 (d, *J* = 7.0 Hz), 6.00, 6.77 (dd, *J* = 8.5, 2.0 Hz), 6.90 (d, *J* = 1.5 Hz), 7.53 (d, *J* = 8.5 Hz); ^{13}C-NMR (C_5D_5N, 125 MHz): 39.9, 55.9, 62.4, 71.3, 74.9, 78.6, 78.8, 102.4, 113.6, 115.6, 116.6, 121.3, 134.3, 138.3, 146.4, 149.9; ESIMS *m*/*z* 349.1 $[M + Na]^+$, 325.0 $[M - H]^-$.

Compound **9**: ^{1}H-NMR (C_5D_5N, 500 MHz): 3.21, 3.76 (3H, s), 3.79 (3H, s), 4.34 (2H), 4.94 (2H), 5.71 (d, *J* = 6.5 Hz), 7.01 (dd, *J* = 8.0, 1.5 Hz), 7.09 (dd, *J* = 8.0, 1.5 Hz), 7.23 (d, *J* = 8.0 Hz), 7.23 (d, *J* = 2.0 Hz), 7.26 (d, *J* = 2.0 Hz), 7.60 (d, *J* = 8.0 Hz); ^{13}C-NMR (C_5D_5N, 125 MHz): 54.7, 54.8, 55.9, 56.0, 62.4, 71.3 (2C), 72.0, 74.9, 78.6, 78.9, 86.3, 86.4, 102.4, 110.9, 111.3, 116.3, 116.5, 119.0, 119.7, 133.0, 136.0, 147.0, 147.9, 148.8, 150.1; ESIMS *m*/*z* 543.2 $[M + Na]^+$, 519.0 $[M - H]^-$.

Compound **10**: ^{1}H-NMR (C_5D_5N, 500 MHz): 3.98, 4.12, 4.28, 4.42 (dd, *J* = 12.0, 5.5 Hz), 4.59 (d, *J* = 11.0 Hz), 4.83 (d, *J* = 12.0 Hz), 4.99 (d, *J* = 7.5 Hz), 5.16 (d, *J* = 12.0 Hz), 7.28 (3H), 7.52 (2H, d, *J* = 7.0 Hz); ^{13}C-NMR (C_5D_5N, 125 MHz): 62.8, 70.8, 71.7, 75.2, 78.5, 78.6, 104.0, 127.7, 128.2 (2C), 128.6 (2C), 138.9; ESIMS *m*/*z* 293.1 $[M + Na]^+$, 269.0 $[M - H]^-$.

Compound **11**: ^{1}H-NMR (C_5D_5N, 500 MHz): 3.36 (2H, d, *J* = 6.5 Hz), 3.75 (3H, s), 5.00 (2H, overlap), 6.05, 6.84 (d, *J* = 8.0 Hz), 6.91 (s), 7.21 (overlap); ^{13}C-NMR (C_5D_5N, 125 MHz): 40.0, 55.8, 113.1, 115.3, 116.5, 121.7, 131.2, 138.8, 146.6, 148.6.

Compound **12**: ^{1}H-NMR (C_5D_5N, 500 MHz): 3.73 (3H),7.26 (d, *J* = 8.0 Hz), 7.57 (overlap), 7.64 (brs), 10.01 (s); ^{13}C-NMR (C_5D_5N, 125 MHz): 55.7, 110.9, 116.5, 127.1, 129.7, 149.2, 154.8, 190.7; ESIMS *m/z* 153.3 $[M + H]^+$, 175.3 $[M + Na]^+$, 151.1 $[M - H]^-$.

Compound **13**: ^{1}H-NMR (C_5D_5N, 500 MHz): 3.88 (3H, s), 3.94 (3H, s), 6.05 (s), 6.93 (d, *J* = 8.0 Hz), 7.55 (d, *J* = 1.5 Hz), 7.63 (dd, *J* = 1.5, 8.0 Hz); ^{13}C-NMR (C_5D_5N, 125 MHz): 51.9, 56.1, 111.7, 114.0, 122.3, 124.2, 146.1, 145.0, 166.9; ESIMS *m/z* 183.3 $[M + H]^+$, 181.1 $[M - H]^-$.

Compound **14**: ^{1}H-NMR (C_5D_5N, 500 MHz): 1.05 (3H, s), 1.05 (3H, s), 1.19, 1.20 (3H, s), 1.46, 1.56, 1.62, 1.81, 1.97, 2.05, 2.20, 2.29, 2.46, 3.73; ^{13}C-NMR (C_5D_5N, 125 MHz): 21.0, 21.2, 27.9, 29.4, 30.8, 34.7, 35.0, 35.1, 36.2, 39.3, 39.9, 43.9, 44.4, 70.1, 71.8; ESIMS *m/z* 256.3 $[M + NH_4]^+$, 221.3 $[M + H - H_2O]^+$, 203.3 $[M + H - 2H_2O]^+$.

Compound **15**: ^{1}H-NMR (C_5D_5N, 500 MHz): 0.92 (3H, s), 1.06 (d, *J* = 12.0 Hz), 1.13 (3H, s), 1.22 (3H, s), 1.22 (overlap), 1.41 (2H), 1.43, 1.59 (dd, *J* = 11.5, 5.0 Hz), 1.89 (d, *J* = 8.0 Hz), 1.95 (bt, *J* = 15.8, 2.5 Hz), 2.14 (tt, *J* = 14.0, 4.0 Hz), 2.21 (d, *J* = 12.5 Hz), 2.51 (td, *J* = 13.5, 5.0 Hz), 3.59 (s), 4.16 (t, *J* = 8.0 Hz); ^{13}C-NMR (C_5D_5N, 125 MHz): 21.3, 25.8, 27.3, 27.9, 29.5, 31.8, 33.8, 35.4, 36.5, 37.3, 45.3, 48.4, 51.2, 74.6, 80.4; ESIMS *m/z* 256.3 $[M + NH_4]^+$.

Compound **16**: ^{1}H-NMR (CD_3OD, 500 MHz): 7.24 (td, *J* = 6.0, 1.0 Hz), 7.28 (td, *J* = 6.0, 1.0 Hz), 7.47 (d, *J* = 8.0 Hz), 8.09 (s), 8.15 (d, *J* = 8.0 Hz), 9.88 (s); ^{13}C-NMR (CD_3OD, 125 MHz): 113.1, 120.2, 122.3, 123.7, 125.1, 125.7, 138.9, 139.5, 187.3; ESIMS *m/z* 146.0 $[M + H]^+$, 143.7 $[M - H]^-$.

Compound **17**: ^{1}H-NMR (C_5D_5N, 500 MHz): 3.77 (3H, s), 6.30 (d, *J* = 9.5 Hz), 7.04 (s), 7.13 (s) , 7.69 (d, *J* = 9.5 Hz); ^{13}C-NMR (C_5D_5N, 125 MHz): 56.2, 104.1, 109.4, 111.0, 112.2, 144.1, 146.3, 151.2, 154.3, 161.5; ESIMS *m/z* 214.9 $[M + Na]^+$, 190.7 $[M - H]^-$.

Compound **18**: ^{1}H-NMR (C_5D_5N, 500 MHz): 3.01 (2H, t, *J* = 7 Hz), 3.88 (m), 6.88 (d, *J* = 15.5 Hz), 7.12–1.26 (7H, m), 8.11 (d, *J* = 15.5 Hz), 8.66 (NH); ^{13}C-NMR (C_5D_5N, 125 MHz): 35.6, 41.9, 55.7, 111.2, 116.3 (2C), 116.8, 119.7, 122.4, 127.4, 130.4 (2C), 130.5, 140.3, 148.8, 149.9, 157.4, 166.7; ESIMS *m/z* 311.9 $[M - H]^-$.

Compound **19**: ^{1}H-NMR (C_5D_5N, 500 MHz): 1.63 (3H, s), 4.10–4.54 (5H); ^{13}C-NMR (C_5D_5N, 125 MHz): 20.6, 63.9, 68.8, 74.7, 76.0; ESIMS *m/z* 159.3 $[M + Na]^+$, 135.3 $[M - H]^-$.

Compound **20**: ^{1}H-NMR (C_5D_5N, 500 MHz): 0.67 (3H, s), 1.21, 1.22, 1.32 (td, *J* = 13.0, 4.0 Hz), 1.51 (3H, s), 1.67 (dt, *J* = 13.0, 3.0 Hz), 1.79 (dt, *J* = 13.0, 2.0 Hz), 1.87 (dd, *J* = 9.5, 4.0 Hz), 1.95, 1.97 (2H), 2.32 (brd, *J* = 10.0 Hz), 2.72 (2H), 3.66, 4.87 (2H, d, *J* = 10.0 Hz), 5.38 (d, *J* = 6.0 Hz), 7.18 (t, *J* = 5.5 Hz); ^{13}C-NMR (C_5D_5N, 125 MHz): 15.2, 23.7, 24.3, 25.0, 29.0, 37.3, 38.2, 39.1, 43.2, 55.3, 56.3, 64.2, 66.0, 75.4, 79.8, 108.8, 130.2, 147.0, 147.9, 170.8; ESIMS *m/z* 373.2 $[M + Na]^+$, 349.1 $[M - H]^-$.

Compound **21**: ^{1}H-NMR (C_5D_5N, 500 MHz): 3.97, 3.99, 4.07 (brt, *J* = 8.1 Hz), 4.30 (2H, m), 4.42 (dd, *J* = 11.7, 5.3 Hz), 4.58 (dd, *J* = 11.8, 2.5 Hz), 5.07 (d, *J* = 7.7 Hz), 5.36 (brs); ^{13}C-NMR (C_5D_5N, 125 MHz): 12.0, 12.2, 19.0, 19.2, 19.4, 20.0, 21.3, 23.4, 24.5, 26.5, 28.6, 29.5, 30.3, 32.1, 32.2, 34.3, 36.4, 37.0, 37.5, 39.4, 40.0, 42.5, 46.1, 50.4, 56.3, 56.9, 62.9, 71.8, 75.4, 78.2, 78.5, 78.6, 102.6, 121.9, 141.0.
